# Harnessing the antioxidant and cytoprotective power of *Aitchisonia rosea*: phytochemical insights and mechanistic validation

**DOI:** 10.1186/s12870-025-07084-7

**Published:** 2025-08-22

**Authors:** Loai Aljerf, Abdullah H. Maad, Shahid Rasool, Muaaz Alajlani

**Affiliations:** 1 Faculty of Pharmacy, Al-Sham Private University, 5910011 Damascus, Syrian Arab Republic; 2 Key Laboratory of Organic Industries, Department of Chemistry, Faculty of Sciences, Damascus University, Damascus, Syrian Arab Republic; 3 Department of Pharmaceutics, Collage of Pharmacy, University of Al- Ameed, Karbala City, Iraq; 4 College of Pharmacy, University of Sargodha, Sargodha, 44100 Pakistan; 5 Faculty of Pharmacy, Arab International University, Damascus, Syrian Arab Republic, Damascus, Syrian Arab Republic

**Keywords:** Cytoprotective, *Aitchisonia rosea*, Oxidative stress, Essential oil, Genotoxicity, DNA integrity, Red blood cell, Natural antioxidant, Phytochemical profiling, Free radical scavenging

## Abstract

**Background:**

*Aitchisonia rosea* (*A. rosea*), traditionally used for oxidative stress-related conditions, lacks comprehensive scientific validation of its antioxidant mechanisms and cellular protective effects. This study aimed to meticulously investigate the phytochemical composition, in vitro antioxidant capacity, and protective efficacy of *A. rosea* extracts and essential oil against oxidative damage in biomolecular and cellular models.

**Results:**

Analysis of the essential oil by gas chromatography-mass spectrometry (GC-MS) identified key bioactive components, including sesquiterpenes (e.g., germacrene, beta-caryophyllene) and oxygenated monoterpenes (e.g., carvacrol, linalool). Various solvent extracts, particularly methanol, ethyl acetate, and *n*-butanol fractions, along with the essential oil, demonstrated potent antioxidant activities across multiple assays including radical scavenging (DPPH), reducing power (FRAP), and Trolox equivalent antioxidant capacity (TEAC), indicating significant free radical neutralisation capabilities. Crucially, these samples provided substantial protection against hydrogen peroxide (H_2_O_2_)-induced genotoxicity, evidenced by reduced DNA damage in comet assays and enhanced preservation of plasmid DNA integrity in gel-based assays. Furthermore, the extracts and essential oil significantly inhibited oxidative haemolysis in red blood cells (RBCs), demonstrating effective membrane stabilisation. Throughout all biological assessments, low cytotoxicity profiles were observed, as demonstrated by the haemolytic assay, affirming the safety of the tested materials.

**Conclusions:**

The findings substantiate the remarkable antioxidant and cytoprotective potential of *A. rosea*, primarily attributed to its rich array of polyphenolic, flavonoid, and terpenoid compounds. The demonstrated ability to safeguard both DNA and erythrocyte membranes from oxidative insults establishes *A. rosea* as a scientifically validated candidate for further pharmaceutical and industrial development, particularly for applications targeting oxidative stress-mediated diseases.

**Supplementary Information:**

The online version contains supplementary material available at 10.1186/s12870-025-07084-7.

## Background

Maintaining cellular homeostasis is a delicate balancing act between the body’s antioxidant defences and the production of reactive oxygen species (ROS) [1]. When this balance is disrupted, oxidative stress ensues, leading to cellular damage and dysfunction. Chronic kidney disease, in particular, has been linked to ROS-mediated damage of red blood cells (RBCs) [2], which has far-reaching implications for various diseases, including cancer, diabetes, cardiovascular disorders, and neurodegenerative diseases [3]. The body’s complex defence mechanisms against oxidative stress underscore the importance of maintaining a balance between ROS production and antioxidant defences [4, 5].

Plants have been recognised as a rich repository of natural antioxidants, which play a vital role in alleviating oxidative stress and its associated detrimental effects [6, 7]. Among these, *Aitchisonia rosea* (*A. rosea*) has garnered significant attention due to its remarkable medicinal properties and potential antioxidant activities [8]. Traditionally used in South and Central Asia, *A. rosea* has been employed in the treatment of a variety of conditions such as fever, gastrointestinal disturbances, inflammation, skin infections, and respiratory issues [9]. Recent pharmacological investigations further support its antimicrobial, anti-inflammatory, analgesic, haemolytic, and anticonvulsant properties [9–11], underscoring its therapeutic versatility and making it a compelling candidate for antioxidant and cytoprotective evaluation. Furthermore, the growing interest in natural products with medicinal properties has led to an increased focus on exploring the antioxidant potential and protective effects of *A. rosea*. As DNA is highly susceptible to oxidative stress, which can induce strand breaks, base modifications, and mutations, compromising genomic stability [12, 13], assessing the capacity of *A. rosea* to shield DNA from oxidative stress is pivotal for understanding its therapeutic potential. This involves investigating the plant’s ability to neutralise ROS and repair oxidative damage, with an emphasis on its effectiveness across a range of temperatures, as thermal variations can influence ROS generation and antioxidant efficacy [14, 15].

Elevated temperatures have been shown to enhance the extraction of bioactive compounds, thereby augmenting the antioxidant potential of the plant [16–20]. However, high temperatures can also lead to the degradation of these compounds, highlighting the need for optimal temperature control during the extraction process [21, 22].

On the other hand, the integrity of RBC membranes is crucial for maintaining proper oxygen transport and overall blood homeostasis [23]. Oxidative stress exacerbates RBC membrane damage, leading to haemolysis and impaired cellular function [23, 24]. The role of *A. rosea* in stabilising RBC membranes under oxidative conditions, particularly in response to temperature-induced stress, is an unexplored yet critical area of research [25].

Phytochemical investigations have revealed the presence of various bioactive compounds, including iridoid glucosides, anthraquinone derivatives, and phenolic compounds [26–29]. While previous studies have demonstrated the anticonvulsant, antimicrobial, and haemolytic effects of *A. rosea* [9, 10, 25], as well as its anti-inflammatory and analgesic activities [9, 11], a significant knowledge gap persists in understanding the unique antioxidant properties of *A. rosea*.

This study aims to address this gap by providing a comprehensive investigation of *A. rosea*’s phytochemical composition, antioxidant capacity, and protective effects against oxidative damage. The scope of this research encompassed extraction, fractionation, and characterisation of bioactive compounds from the plant material, as well as assessment of their efficacy in protecting DNA and RBCs from oxidative insult. By shedding light on the mechanisms underlying *A. rosea*’s antioxidant and cytoprotective properties, this study contributes to the growing body of knowledge on natural antioxidants and supports the potential of *A. rosea* as a therapeutic candidate in oxidative stress-related pathologies.

## Materials and methods

### Collection of plant specimens

The plant material used in this investigation was *A. rosea*, collected during its flowering stage in mid-April 2024, a period corresponding to the spring bioclimatic stage in the hilly regions of Quetta, Baluchistan, Pakistan [30]. The timing of the collection ensured optimal phytochemical content, as bioactive compound concentrations are often influenced by the plant’s phenological stage and environmental factors. The specimens were rigorously identified and authenticated by the taxonomist Dr. Shahid Rasool, and a voucher specimen (No. 1911) was deposited in the Sultan Ayoub Herbarium, GC University Lahore, for future reference. The botanical nomenclature was cross-verified using the online database http://www.theplantlist.org to ensure taxonomic accuracy.

### Extraction and fractionation of bioactive compounds from plant material

The preparation of the methanol extract and its fractions involved a series of meticulously executed steps to ensure the efficient extraction of bioactive compounds:

### Preparation of plant material

The stems and branches of *A. rosea* were collected, arranged on laboratory tables, and allowed to shade dry for seven days under ambient conditions (25 ± 2 °C, relative humidity 45–50%). This step facilitated the removal of moisture while minimising potential degradation of sensitive phytochemicals. The dried plant material was pulverised using an electric mill (Fritsch Pulverisette 14, Fritsch GmbH, Idar-Oberstein, Germany) to produce a fine powder with a particle size of 0.5 mm, which was subsequently stored in airtight containers under desiccated conditions until further processing.

### Extraction and fractionation procedures

To prepare the methanol extract, 10 kg of powdered plant material was soaked in methanol (3 × 12 L; analytical grade, purity ≥ 99.8%, Sigma-Aldrich, St. Louis, MO, USA) for seven days at room temperature (25 ± 2 °C) with occasional stirring to enhance solubilisation of bioactive compounds. Following each cycle, the mixture was filtered through Whatman No. 1 filter paper (GE Healthcare, Maidstone, UK), and the filtrates were pooled. The combined methanol extracts were concentrated to dryness under reduced pressure using a rotary evaporator (Heidolph, model Laborata 4000, Schwabach, Germany) at 40 °C, ensuring complete removal of the solvent. The resulting dried methanol extract (yield: 27.0% ± 1.5%, 95% confidence limit (CL), *n* = 3) was weighed and stored in a vacuum desiccator for subsequent fractionation.

The dried methanol extract was subjected to successive solvent partitioning to fractionate the extract based on polarity. The extract (500 g) was sequentially dissolved in distilled water and partitioned with ethyl acetate, *n*-butanol, chloroform, and *n*-hexane (Merck analytical grade, purity ≥ 99.5%, Merck KGaA, Darmstadt, Germany) in a separatory funnel. Each solvent partitioning step was repeated thrice to ensure maximal recovery of compounds.

The organic layers obtained during each step were pooled and evaporated to dryness under reduced pressure using a rotary evaporator at a temperature specific to each solvent (*n*-butanol: 50 °C, ethyl acetate: 45 °C, chloroform: 40 °C, and *n*-hexane: 35 °C). The resultant fractions were weighed to determine their respective yields:

- *n*-Butanol fraction: 8.0% ± 0.8% (95% CL, *n* = 3).
- Ethyl acetate fraction: 6.0% ± 0.6% (95% CL, *n* = 3).
- Chloroform fraction: 5.4% ± 0.7% (95% CL, *n* = 3).
- *n*-Hexane fraction: 3.0% ± 0.5% (95% CL, *n* = 3).

Each fraction was stored in amber glass vials at 4 °C to protect them from light and oxidation, ensuring their stability for subsequent analysis.

The successive solvent extraction method was chosen to facilitate selective recovery of bioactive compounds based on polarity gradients. This approach is widely recognised in phytochemistry for its efficacy in isolating diverse compounds [26–31]. By employing this method, the study ensured the targeted extraction of hydrophobic, semi-polar, and polar phytochemicals, enabling a comprehensive analysis of the plant’s phytochemical composition.

### Phytochemical profiling

A thorough phytochemical analysis was conducted to identify and quantify the diverse array of bioactive compounds present in the methanol extract and its subsequent fractions (ethyl acetate, *n*-butanol, chloroform and *n*-hexane). Employing established methodologies, the presence of various biochemical classes was systematically investigated [32, 33].

### Essential oil isolation and characterisation

The extraction of essential oil from plant material was carried out via hydro-distillation, following established protocols [34, 35]. The resulting essential oil was collected, and the percentage yield was calculated.

To facilitate the analysis of the essential oil, a gas chromatography (GC) 6850 network system equipped with a 7683B series auto-injector and 5973 inert mass detector (Agilent Technologies, Wilmington, DE, USA) was utilised. An HP-5 MS capillary column with a 5% phenyl polysiloxane stationary phase (30.0 m × 0.25 mm, film thickness 0.25 μm) was employed to separate the compounds.

The oven temperature (OT) was initially maintained at 45 °C for 5 min, then increased to 150 °C at a rate of 10 °C per minute. Subsequently, OT was increased to 280 °C at a rate of 5 °C per minute and finally, OT was increased to 325 °C at a rate of 15 °C per minute and maintained at this temperature for 5 min. Helium gas was flown at a pressure of 60 kPa and linear velocity of 38.2 cm/sec at a rate of 1.1 mL/min for monitoring the components in scanning mode from 40 to 550 m/z [36]. For this analysis, a sample size of 10 µL was used, with a CL of 95% and a precision of ± 0.5%. The gas chromatography-mass spectrometry (GC-MS) analysis was performed in triplicate, with a retention time (RT) repeatability of ± 0.05 min. The identification of the essential oil compounds was based on the comparison of their mass spectra with those of the National Institute of Standards and Technology (NIST) 05 library, with a similarity index ≥ 80%. The relative percentage of each compound was calculated based on the peak area normalisation method.

The validation of the GC-MS method was performed according to the International Conference on Harmonisation (ICH) guidelines for validation of analytical procedures [37]. The method was found to be linear over a range of 0.1–100 µg/mL, with a coefficient of determination (R²) of 0.999. The limit of detection (LOD) and limit of quantitation (LOQ) were determined to be 0.05 µg/mL and 0.15 µg/mL, respectively. This protocol provides a comprehensive and reliable approach for the extraction and analysis of essential oils from plant material, ensuring the accuracy and precision of the results.

### Determination of total phenolic content (TPC) using modified Folin-Ciocalteu colorimetric method

The total phenolic content (TPC) of the plant material was accurately determined using a modified version of the Folin-Ciocalteu colorimetric method, as described by Chaovanalikit and Wrolstad [38]. This method is widely recognised for its reliability and reproducibility in quantifying phenolic compounds in plant extracts.

A 100 µL aliquot of each extract and fraction (1 mg/mL in methanol) was carefully dissolved in 7.5 mL of distilled water to create a homogeneous solution. This step ensured that the phenolic compounds were evenly distributed and readily available for reaction with the Folin-Ciocalteu reagent (Phosphomolybdic acid (PMA) and phosphotungstic acid (PTA); Sigma-Aldrich, St. Louis, MO, USA). Subsequently, 0.5 mL of Folin-Ciocalteu reagent was added to the mixture, which was then incubated at room temperature (20 °C ± 2 °C) for 10 min. This allowed for the formation of a blue-coloured complex between the phenolic compounds and the Folin-Ciocalteu reagent. Following the incubation period, 1.5 mL of 20% (w/v) sodium carbonate (Na_2_CO_3_) (Thermo Fisher Scientific, Waltham, MA, USA) was added to the mixture, which was then heated to 40 °C for 20 min using a water bath. This step facilitated the reduction of the blue-coloured complex, resulting in the formation of a stable chromophore. After heating, the mixture was cooled rapidly using an ice bath to stop the reaction. The resulting solution was then used for absorbance measurement at 725 nm using a spectrophotometer (UV-1800, Shimadzu, Kyoto, Japan). A control solution was prepared by omitting the extracts and fractions from the above mixture, following the same protocol as described above. This control solution served as a blank for the spectrophotometric analysis.

A calibration curve for gallic acid (GA) (Sigma-Aldrich, St. Louis, MO, USA) was constructed using linear dose-response regression analysis on an Excel spreadsheet, with concentrations of 10, 20, 40, 80, 100, and 120 µg/mL (Fig. S1). The TPC was expressed as milligrams of GA equivalents (GAE) per gram of sample, with a CL of 95% and a precision of ± 2%. This method has been demonstrated to be reliable and reproducible, with a CV of less than 5% [39].

The use of GA as a standard has been widely accepted and validated in previous studies [40], making it an ideal choice for TPC determination. The Folin-Ciocalteu method has been extensively used for the determination of TPC in various plant extracts, and its reliability and accuracy have been well-established.

### Total flavonoid content (TFC) determination

The total flavonoid content (TFC) of *A. rosea* extracts was determined using the aluminium chloride (AlCl_3_) colorimetric method, which is based on the formation of a stable complex between AlCl_3_ and the carbonyl and hydroxyl groups of flavonoids, resulting in a measurable colour change [41, 42]. Analytical grade reagents were used, including AlCl_3_, sodium hydroxide (NaOH), sodium nitrite (NaNO_2_), and quercetin (Sigma-Aldrich, St. Louis, MO, USA) as the standard. The extracts were dissolved in a suitable solvent (e.g., methanol or ethanol). A standard curve was prepared using various concentrations of quercetin (e.g., 0–100 µg/mL). Dilutions of the standard were used to establish a linear relationship between concentration and absorbance.

Briefly, 0.5 mL of each *A. rosea* extract solution (or quercetin standard) was mixed with 0.5 mL of 5% NaNO_2_ solution. The mixture was allowed to stand for 5 min at room temperature. Subsequently, 0.5 mL of 10% AlCl_3_ solution was added, and the mixture was allowed to stand for an additional 6 min. Finally, 2.0 mL of 1 M NaOH solution was added, and the total volume was immediately made up to 5 mL with distilled water. The mixture was then thoroughly mixed, and its absorbance was measured at 510 nm using a spectrophotometer (Shimadzu UV-1800, Kyoto, Japan) against a reagent blank. The TFC of the extracts was expressed as milligrams of quercetin equivalents per gram of extract (mg QE/g extract). This was determined by extrapolating the absorbance values of the samples against the established quercetin standard curve. All measurements were performed in triplicate, and the results are reported as the mean ± standard deviation (SD).

### Antioxidant activity assays

The antioxidant capacity of the plant methanol extract, its various organic fractions, and essential oil was assessed using two assays: the 2,2-diphenyl-1-picrylhydrazyl (DPPH) free radical scavenging assay and the ferric reducing antioxidant power (FRAP) assay [43, 44].

### Antioxidant capacity assessment using DPPH free radical scavenging assay

To evaluate the antioxidant potential of the plant methanol extract, its various organic fractions, and essential oil, we employed the DPPH free radical scavenging assay, a well-established method for assessing the antioxidant capacity of test samples [45]. This assay enables the quantification of the scavenging activity of antioxidants against DPPH radicals, which are stable free radicals that can be reduced by antioxidants, resulting in a decrease in absorbance.

The experimental procedure involved adding 10 µL of each test solution, prepared at various concentrations ranging from 15 to 500 µg/mL, to individual wells of a 96-well plate. This was followed by the addition of 90 µL of 100 µM methanol DPPH (2,2-diphenyl-1-picrylhydrazyl) (Sigma-Aldrich, St. Louis, MO, USA) solution, resulting in a total volume of 100 µL. The plate was then incubated at 37 °C for 30 min to facilitate the reaction between the test samples and DPPH radicals. Subsequently, the absorbance was measured at 517 nm using a microplate reader (Thermo Fisher Scientific, Waltham, MA, USA) to quantify the reduction of DPPH radicals.

Ascorbic acid (AA) (Alfa Aesar, Haverhill, MA, USA), referred later to as Vitamin C in Table 3, served as a reference antioxidant agent and was tested at the same concentration range as the extracts (15 to 500 µg/mL) to allow for direct comparison.

To minimise experimental errors, triplicate experiments were performed for each test sample. The half-maximal inhibitory concentration (IC₅₀) values were determined using extract concentrations of 500, 250, 125, 62.5, 31.3, and 15.0 µg/mL. Data analysis was performed using the Ez-fit software (Perella Scientific Inc., Amherst, USA). The decrease in absorbance value indicated an enhanced radical scavenging mechanism, calculated using the following equation:

1 $${\text{Inhibition }}\left( \% \right) = \left( {{\text{Ac }}{-}{\text{ As}}} \right)/{\text{ Ac }} \times {\text{100}}$$

where Ac represents the absorbance of the control and As represents the absorbance of the sample. Equation 1 enables the calculation of the percentage inhibition of DPPH radicals by the test samples, providing a quantitative measure of their antioxidant capacity.

### Determination of antioxidant capacity by ferric reducing antioxidant power (FRAP) assay

To comprehensively evaluate the antioxidant capacity of the test samples, the FRAP assay was employed, based on the well-established method described by Benzie and Strain [43] and modified according to Pulido et al. [46]. This widely accepted and reliable method has been extensively utilised for assessing the antioxidant activity of various compounds, making it an ideal choice for evaluating the test samples. The FRAP assay is predicated on the reduction of ferric ions (Fe^3+^) by antioxidants, resulting in the formation of ferrous ions (Fe^2+^), which can then react with FeCl_3_ to produce a coloured complex. The intensity of the resulting colour is directly proportional to the reducing power of the antioxidants present in the sample [43].

To ensure the accuracy and reliability of the results, a thorough sample preparation protocol was implemented. Specifically, thirty replicate samples (*n* = 30) of each test material, including the plant methanol extract, fractions (ethyl acetate, *n*-butanol, chloroform and *n*-hexane), and essential oil, were prepared at concentrations ranging from 62.5 to 1000 µg/mL in 1 mL of deionised water. AA, referred to as Vitamin C in Table 3; Fig. 2, was used as a positive control and was tested at the same concentration range as the extracts (62.5 to 1000 µg/mL). This concentration range for the FRAP assay, which differs from that used in the DPPH assay, was specifically chosen to ensure the absorbance values remained within the spectrophotometer’s linear detection range for the FRAP reaction kinetics.

The prepared solutions were then mixed with 2.5 mL of 200 mM sodium phosphate buffer (pH 6.6, prepared from disodium phosphate (Na_2_HPO_4_) and monosodium phosphate (NaH_2_PO_4_); Merck, Darmstadt, Germany) and 1% potassium ferricyanide (K_3_[Fe(CN)_6_]) (Acros Organics, Geel, Belgium), which facilitated the reduction of Fe^3+^ ions. The resulting mixture was incubated at 50 °C for 20 min to allow for the reduction of Fe^3+^ ions to occur. Subsequently, 2.5 mL of 10% trichloroacetic acid (TCA, CCl_3_COOH) (Carl Roth, Karlsruhe, Germany) was added to the mixture, and it was centrifuged at 3000 rpm for 5 min to separate the phases. This step was crucial in precipitating the proteins and other interfering compounds, thereby ensuring that the subsequent colorimetric measurement was not compromised. Following centrifugation, the upper layer was removed, and an aliquot volume (2.5 mL) from the supernatant layer was added to 3 mL of a solution containing distilled water and 0.1% FeCl_3_ (Sigma-Aldrich, St. Louis, MO, USA) (1:2 v/v). The absorbance of this solution was measured at 700 nm using a UV-visible spectrophotometer. The absorbance values were recorded, and the results are expressed as mean ± SD of the absorbance values. Moreover, to ensure the validity and reliability of the results, a stringent data analysis protocol was implemented. The CLs were set at 95% (*p* < 0.05) to guarantee the accuracy of the results. The precision of the assay was determined by calculating the coefficient of variation (CV) for each sample, which provided an indication of the reproducibility of the results. According to Chen et al. [47], higher absorbance values correspond to higher reducing power of the analysed samples, indicating a greater antioxidant capacity. By following this protocol, the FRAP assay provides a robust and reliable method for evaluating the antioxidant capacity of various test samples, enabling the identification of potent antioxidants and their potential applications in various fields.

Overall, the FRAP assay offers a sensitive and reliable method for assessing the antioxidant capacity of various compounds, making it an essential tool in the evaluation of the test samples. The results obtained from this assay can be used to inform the development of novel antioxidant-based therapies and products, which can have a significant impact on human health and well-being.

### DNA protection assay: methodology and experimental design

The purpose of this experiment was to investigate the protective effects of plant extracts on DNA damage using a modified adaptation of the previously described approach [48]. To achieve this, a comprehensive experimental design was employed, involving the treatment of plasmid pBR 322 DNA with against hydrogen peroxide (H_2_O_2_) and ultraviolet (UV) light as a control. To prepare the reaction mixture, 0.5 µg of plasmid pBR 322 DNA (Thermo Fischer Scientific, Waltham, MA, USA) was diluted with 3 µL of 50 mM sodium phosphate buffer (pH 7.4, prepared from Na_2_HPO_4_ and NaH_2_PO_4_; Merck, Darmstadt, Germany) to create a stable environment for DNA interaction. Subsequently, the mixture was treated with varying concentrations (10, 100, and 1000 µg/mL) of plant methanol extract to assess the dose-dependent effects on DNA protection. Notably, the most pronounced protective effect on DNA was observed at a concentration of 1000 µg/mL, which was further processed for the organic fractions and essential oil.

To induce oxidative stress, 4 µL of 30% H_2_O_2_ (Merck, Darmstadt, Germany), a concentration known to be toxic to DNA, was added to the reaction mixture. The mixture was then incubated in the dark at 37 °C for 1 h to allow for optimal DNA-H_2_O_2_ interaction. This incubation period enabled the evaluation of the plant extract’s protective effects on DNA against oxidative damage. Following incubation, the plasmid DNA was resolved on a 1% agarose (Invitrogen, Carlsbad, CA, USA) gel, prepared by dissolving 1 g of agarose in 100 mL of Tris-Borate-ethylenediaminetetraacetic acid (TBE-EDTA) buffer (prepared from Tris base, boric acid (H_3_BO_3_), and EDTA; AppliChem, Darmstadt, Germany).

Electrophoresis was performed at 100 volts for 1 h using an electrophoresis apparatus (Bio-Rad Laboratories, Hercules, CA, USA), allowing for the separation of native and oxidised DNA. The migration pattern of native DNA was compared to that of oxidised DNA to determine the difference in DNA integrity [49].

A total of 12 samples were analysed in triplicate, with a sample size of 3 (*n* = 3), to ensure robust and reliable results. The CLs were set at 95% (*α* = 0.05), and the precision was ± 5%. The results are presented as the mean ± SD. The DNA protection assay was repeated three times, and the results were consistent across all replicates.

By employing this comprehensive methodology, the present study aimed to provide a detailed understanding of the protective effects of plant extracts on DNA damage, shedding light on their potential applications in the prevention and treatment of oxidative stress-related diseases.

### Determination of DNA binding affinity

The interaction and binding affinity of *A. rosea* extracts and essential oil with DNA were quantitatively assessed using both UV-Visible absorption spectrophotometry and fluorescence spectroscopy. These complementary spectroscopic techniques allow for the determination of the equilibrium dissociation constant (K_D_​), providing insight into the strength of the molecular interaction between the test compounds and DNA. All measurements were conducted at 25 °C.

Calf thymus DNA (CT-DNA, Sigma-Aldrich, St. Louis, MO, USA) was employed as a model for DNA. A stock solution of CT-DNA was prepared by dissolving it in 50 mM Tris-hydrochloric acid (HCl) buffer (pH 7.4, prepared from Tris(hydroxymethyl)aminomethane (Tris base, NH_2_C(CH_2_OH)_3_) and HCl; Merck, Darmstadt, Germany) to a final concentration of 1 mM. The exact concentration of the CT-DNA stock solution was precisely determined spectrophotometrically at 260 nm, utilising a molar extinction coefficient of 6600 M^−1^cm^−1^ [50]. The purity of the DNA preparation was rigorously verified by assessing the ratio of absorbances at 260 nm and 280 nm (A260​/A280​), which was consistently maintained between 1.8 and 1.9, indicating minimal protein contamination. *A. rosea* extracts and essential oil were prepared as concentrated stock solutions at 1 mg/mL in dimethyl sulfoxide (DMSO; Merck, Darmstadt, Germany) and subsequently diluted with the same 50 mM Tris-HCl buffer (pH 7.4) to achieve the desired working concentrations for the assays.

UV-Visible absorption spectra were recorded using a Shimadzu UV-1800 spectrophotometer. For each *A. rosea* extract or essential oil, a fixed concentration of 50 µM was prepared. These solutions were then titrated by the incremental addition of increasing concentrations of CT-DNA, ranging from 0 to 500 µM. Absorbance changes were meticulously monitored across the wavelength range of 200–400 nm. To isolate the absorbance changes due to complex formation, the intrinsic absorption of CT-DNA at each corresponding concentration was subtracted from the spectra of the compound-DNA mixtures. The K_D_ constants were determined by plotting the observed change in absorbance as a function of the added DNA concentration. The data were then fitted to a non-linear regression model assuming a 1:1 binding stoichiometry using GraphPad Prism 9.0 software (San Diego, CA, USA).

Fluorescence emission spectra were recorded using a PerkinElmer LS 55 fluorescence spectrophotometer (Waltham, MA, USA). The competitive binding of *A. rosea* compounds with DNA was assessed using Ethidium Bromide (EtBr, Sigma-Aldrich, St. Louis, MO, USA) as a fluorescent probe. A solution containing 10 µM EtBr and 100 µM CT-DNA was prepared and allowed to equilibrate. Increasing concentrations of *A. rosea* extracts or essential oil (ranging from 0 to 200 µg/mL or 0–50 µM, depending on the sample’s potency) were then incrementally added to the pre-formed DNA-EtBr solution. The fluorescence emission was monitored at 600 nm upon excitation at 520 nm. The observed quenching of EtBr fluorescence, indicative of its displacement from the DNA, was analysed using the modified Stern-Volmer equation (Eq. 2) for competitive binding [51]:

2 $$\:\frac{{F}_{0}}{F}=1+{K}_{sv}\left[Q\right]$$

where F_0_​ and F are the fluorescence intensities in the absence and presence of the quencher (extract/essential oil), respectively, K_sv_​ is the Stern-Volmer quenching constant, and [Q] is the concentration of the quencher. The K_D_​ values were subsequently derived from the quenching constants using established relationships for competitive binding, as implemented in GraphPad Prism 9.0. All experiments for DNA binding affinity were performed in triplicate. Results are expressed as the mean ± SD of these independent experiments.

### Hydroxyl radical scavenging activity of extracts: a deoxy-D-ribose degradation assay

This assay was employed to evaluate the hydroxyl radical (OH·) scavenging activity of the extracts. This assay measures the ability of the extracts to inhibit the degradation of 2-deoxy-D-ribose, a sensitive indicator of OH·**-**mediated damage.

The reaction mixture consisted of 2.5 mM 2-deoxy-D-ribose (Carl Roth, Karlsruhe, Germany), 100 µM FeCl_3_, 100 µM EDTA, 100 µM AA, and 1 mM H_2_O_2_ in a phosphate buffer solution with a pH of 7.4 [49]. Extract samples were added to the reaction mixture at a final concentration of 1 mg/mL. The mixture was then incubated at 37 °C for 1 h to allow for the reaction to occur. Following incubation, the extent of 2-deoxy-D-ribose degradation was quantified using the thiobarbituric acid reactive substances (TBARS) method. The OH^•^ radical scavenging activity of the extracts was expressed as the percentage inhibition of 2-deoxy-D-ribose degradation relative to a control reaction without the extract. This percentage inhibition value represents the ability of the extract to scavenge OH^•^ radicals and prevent 2-deoxy-D-ribose degradation. By employing this assay, the OH^•^ radical scavenging activity of the extracts can be accurately quantified, providing valuable insights into their antioxidant properties and potential applications.

### Trolox equivalent antioxidant capacity (TEAC) assay

The Trolox Equivalent Antioxidant Capacity (TEAC) assay was performed to evaluate the free radical scavenging activity of *A. rosea* extracts, based on their ability to decolourise the 2,2′-azino-bis(3-ethylbenzothiazoline-6-sulfonic acid) (ABTS·+) radical cation [52]. This spectrophotometric method measures the reduction of the pre-formed ABTS·+ radical by antioxidants, resulting in a decrease in absorbance at 734 nm.

ABTS (Sigma-Aldrich, St. Louis, MO, USA) and potassium persulphate (K_2_S_2_O_8_) (Sigma-Aldrich, St. Louis, MO, USA) were used for ABTS•+ radical generation. Trolox (6-hydroxy-2,5,7,8-tetramethylchroman-2-carboxylic acid, Sigma-Aldrich, St. Louis, MO, USA), a water-soluble vitamin E analog, was used as the standard. Phosphate-buffered saline (PBS, pH 7.4) was prepared using analytical grade reagents.

The ABTS•+ radical cation solution was prepared by reacting 7 mM ABTS solution with 2.45 mM K_2_S_2_O_8_ solution. The mixture was allowed to stand in the dark at room temperature for 12–16 h to ensure complete formation of the radical. Before use, the ABTS·+ solution was diluted with PBS (pH 7.4) to an absorbance of 0.70 ± 0.02 at 734 nm, measured using a Shimadzu UV-1800 spectrophotometer.

Briefly, 10 µL of each *A. rosea* extract (at concentrations of 10, 25, 50, 100, 200 µg/mL) or Trolox standard solution (at concentrations of 5, 10, 20, 40, 80 µM) were added to 990 µL of the diluted ABTS·+ solution. The mixture was vigorously shaken and incubated in the dark at room temperature for 6 min. The absorbance was then measured at 734 nm against a blank sample (PBS). A standard curve was generated using different concentrations of Trolox (0-500 µM).

The percentage of ABTS•+ radical scavenging was calculated using the following formula (Eq. 3) [53]:

3 $$\% {\text{ Scavenging }} = {\text{ }}\left[ {\left( {{\text{A}}_{0} {\text{ }} - {\text{ A}}_{1} } \right)/{\text{A}}_{0} } \right]{\text{ }} \times {\text{100}}$$

where A₀ is the absorbance of the ABTS·+ radical solution without the sample, and A₁ is the absorbance of the ABTS·+ radical solution with the sample.

The TEAC value of each extract was expressed as micromoles of Trolox equivalents per gram of extract (µmol TE/g extract), calculated from the Trolox standard curve. All experiments were performed in triplicate, and results were expressed as mean ± SD.

### Evaluation of red blood cell (RBC) membrane protection against oxidative stress

We employed a modified haemolytic assay, as described in previous research [54–57], to evaluate the cytoprotective potential of the crude extract, subsequent fractions, and essential oil of the test plant against oxidative stress-induced damage to RBC membranes. This investigation was conducted in accordance with the ethical guidelines set forth by the College of Pharmacy, University of the Punjab, Lahore, Pakistan, ensuring the highest standards of research integrity.

Fresh human blood samples were obtained from healthy adult volunteers who provided written informed consent prior to participating in the study. The rights and confidentiality of all participants were protected throughout the research process, in adherence to the principles of the Declaration of Helsinki and guidelines for the care and use of laboratory humans. RBCs were isolated and processed according to established protocols to assess the protective effects of *A. rosea* extracts under oxidative stress conditions. By utilising this modified haemolytic assay, we aimed to investigate the potential of the test plant’s bioactive compounds to safeguard RBC membranes against oxidative stress, thereby shedding light on the therapeutic potential of this plant in mitigating oxidative stress-related disorders.

### Assessment of erythrocyte membrane cytotoxicity (haemolytic assay)

In accordance with the guidelines set forth by the College of Pharmacy, University of the Punjab, Lahore, Pakistan, and approved by the institutional ethical committee, a modified haemolytic assay was employed to evaluate the cytotoxic potential of the crude extract, subsequent fractions, and essential oil of the test plant, as described by Aslam et al. [54].

### Blood sample collection and preparation

A total of 30 healthy human blood samples, comprising 15 male and 15 female volunteers, were collected after obtaining informed consent. RBCs were isolated from the blood samples through centrifugation, followed by triple washing with chilled sterile isotonic phosphate-buffered saline (PBS) solution, pH 7.4. The erythrocyte count was standardised to 7.068 × 10⁸ cells/mL for each test.

### Assay procedure

In Eppendorf tubes, 20 µL of each test substance (1 mg/mL in DMSO) was combined with 180 µL of diluted RBC suspension. The positive and negative controls consisted of 0.1% Triton X-100 (Sigma-Aldrich, St. Louis, MO, USA) and PBS, respectively. Following incubation at 37 °C for 35 min, the tubes were transferred to an ice bath for 5 min, and then centrifuged for 5 min. The resulting supernatant (100 µL) was diluted with 900 µL of chilled PBS solution in Eppendorf tubes, which were then placed in an ice bath. Subsequently, 200 µL of the mixture from each tube was transferred to 96-well microtiter plates. The experiments were performed in triplicate.

### Absorbance measurement and data analysis

Absorbance was measured at 576 nm using a Bio-Tek EL404 uQuant Universal Microplate Spectrophotometer (Artisan TG, 101 Mercury Drive, Champaign, IL, USA). The percentage of RBC lysis was calculated using the Eq. 4 [58]:

4 $${\text{RBC lysis }}\left( \% \right) = \left( {{\text{As }}/{\text{ At}}} \right)\times {\text{100}}$$

where As and At represent the absorbances of the sample and Triton X-100, respectively.

#### Statistical analysis

The findings of this study are presented with a high degree of precision, expressed as mean values accompanied by their corresponding standard errors (SE). To establish statistically significant differences between the groups, an analysis of variance (ANOVA) was initially conducted, followed by Duncan’s multiple range test (DMRT) as a post-hoc analysis to identify specific pairwise differences. A significance level of *p* < 0.05 was adopted, which corresponds to a 95% CL. The sample size calculation was informed by the requirement to achieve a power of 0.8 at an alpha level of 0.05, with a minimum of 10 replicates per group. The margin of error was set at ± 10% to quantify the precision of the estimates. Statistical analyses were performed using the Statistical Package for the Social Sciences (SPSS) software (version 25.0; IBM Corp., Armonk, NY, USA). The normality of the data was evaluated using the Shapiro-Wilk test, while the homogeneity of variance was assessed using Levene’s test. In instances where the assumptions of normality or equal variances were not met, non-parametric tests (Kruskal-Wallis’s test and Mann-Whitney U test) were employed to ensure the robustness and validity of the results.

## Results

The present study aimed to comprehensively investigate the phytochemical composition, antioxidant activities, and protective effects of *A. rosea* essential oil and various solvent extracts against oxidative stress-induced damage. The detailed findings are presented below.

### Chemical composition of *A. rosea* essential oil

The essential oil extracted from *A. rosea* exhibited a yield of 0.27% (w/w dry plant material). GC-MS analysis identified a diverse array of bioactive compounds, collectively accounting for 99.9% of the total oil composition (Table 1, Fig. S2). The most abundant constituents were germacrene (18.43 ± 0.50%), octadecanoic acid methyl ester (14.43 ± 0.45%), and carvacrol (12.80 ± 0.32%). Other significant components included *β*-caryophyllene (5.89 ± 0.19%), linalool (2.67 ± 0.12%), and *β*-elemene (2.66 ± 0.13%). These compounds have been previously documented for their diverse biological activities, including antioxidant and protective effects [57].

Further statistical analyses elucidated relationships within the essential oil’s chemical profile. Pearson’s correlation coefficients revealed a strong positive correlation between *β*-caryophyllene and germacrene (*r* = 0.83, *p* < 0.05), suggesting a potential common biosynthetic or metabolic pathway. A moderate positive correlation was also observed between linalool and carveol (*r* = 0.56, *p* < 0.05), indicating potential shared functional or biosynthetic roles. Principal Component Analysis (PCA) identified major patterns in the data, with the first two principal components explaining 44.1% and 65.6% of the total variance, respectively. PC1 was predominantly influenced by carvacrol, germacrene, and *β*-caryophyllene compounds recognised for their antimicrobial and antioxidant properties [59]. PC2 was characterised by linalool, carveol, and *α*-muurolol, which are often associated with sedative activities. Hierarchical Cluster Analysis (HCA) further categorised the compounds into three distinct groups based on their relative abundances: Cluster 1 (high abundance: carvacrol, germacrene, and *β*-caryophyllene), Cluster 2 (moderately abundant: linalool, carveol, and *α*-muurolol), and Cluster 3 (low abundance: *α*-pinene, camphene, and myrcene). These groupings potentially reflect distinct biosynthetic pathways or functional roles of these compounds within *A. rosea*.

**Table 1:** GC-MS identified compounds in *A. rosea* essential oil

Compound	Mean Area (%) ± SEM	Retention Index (RI)	Group (Tukey’s HSD)
*Monoterpenes & Oxygenated Derivatives*			
*α*-Pinene	0.79 ± 0.03	940	c
Camphene	2.48 ± 0.10	953	b
*β*-Pinene	1.27 ± 0.05	970	c
Myrcene	0.73 ± 0.02	979	c
Cymene	1.50 ± 0.04	1025	c
Limonene	0.65 ± 0.03	1028	c
Cineole	1.76 ± 0.06	1030	c
*β-*Phellandrene	0.78 ± 0.03	1033	c
Linalool	2.67 ± 0.12	1098	b
Carveol	0.80 ± 0.03	1138	c
Verbenone	0.98 ± 0.04	1204	c
Thymol	2.96 ± 0.15	1291	b
Undecanone	0.73 ± 0.03	1293	c
Carvacrol	12.80 ± 0.32	1297	a
*Sesquiterpenes & Related Compounds*			
*β*-Elemene	2.66 ± 0.13	1392	b
Eugenol	2.33 ± 0.11	1411	b
*α*-Copaene	1.72 ± 0.08	1411	c
*β*-Caryophyllene	5.89 ± 0.19	1417	b
Germacrene	18.43 ± 0.50	1483	a
Valencene	2.78 ± 0.13	1493	b
Cadinene	3.02 ± 0.15	1513	b
Spathulenol	3.66 ± 0.17	1577	b
*α*-Muurolol	3.75 ± 0.19	1647	b
*α*-Cadinol	1.43 ± 0.06	1654	c
*Fatty Acid Esters & Hydrocarbons*			
Hexadecanoic acid methyl ester	1.82 ± 0.08	1962	c
Eicosane	2.35 ± 0.11	2000	b
Octadecanoic acid methyl ester	14.43 ± 0.45	2124	a
Total identified compounds (%)	99.9 ± 0.02		-
Different letters in the Statistical Grouping column indicate statistically significant differences (*p* < 0.05) based on Tukey’s HSD test			

### Total phenolic and flavonoid contents

**Fig. 1:**
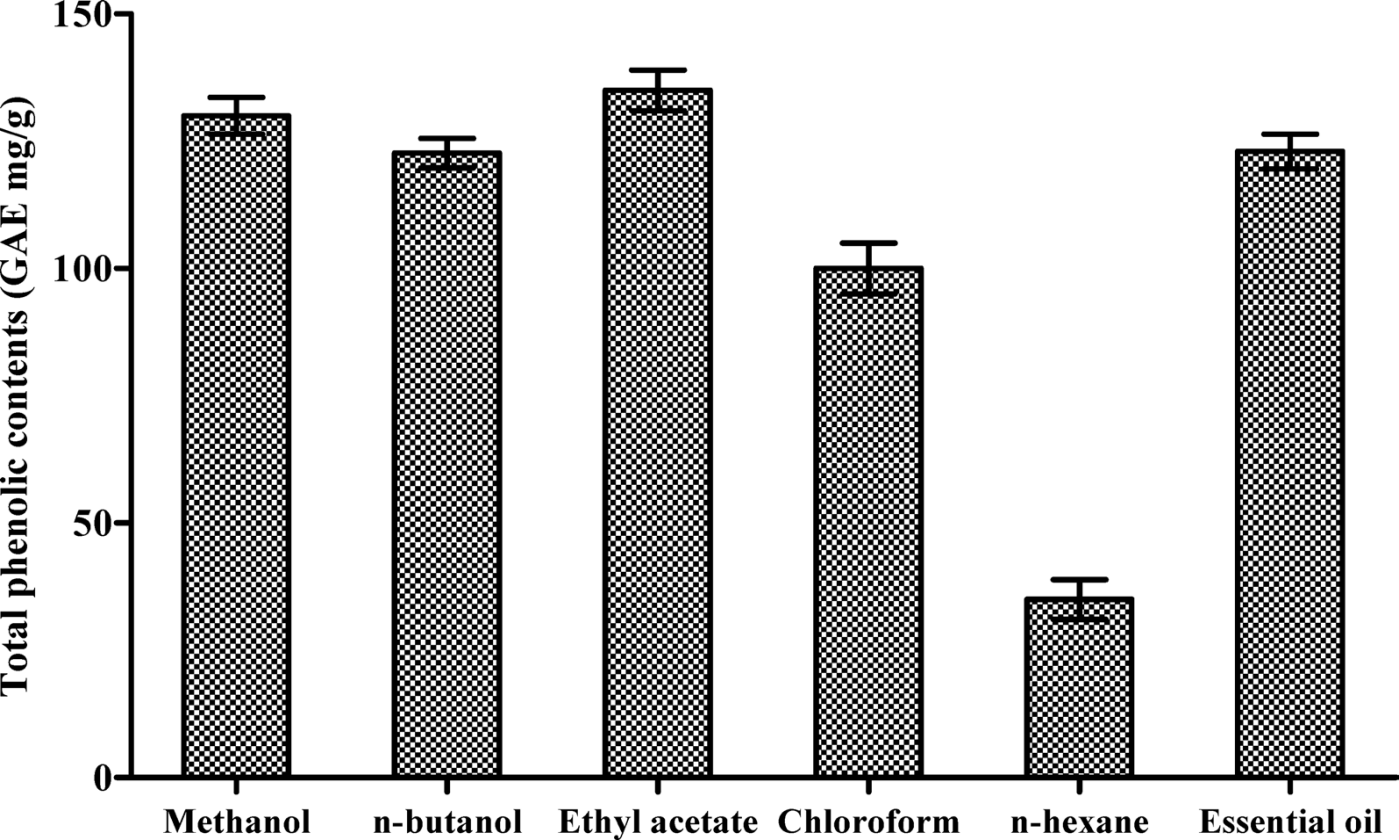
Comparative analysis of total phenolic contents in *A. rosea* organic solvent extracts and essential oil extract

The TPC and TFC were determined for the *A. rosea* extracts and essential oil (Table 2). The TPC analysis (Fig. 1) revealed considerable variation among the tested samples. The ethyl acetate exhibited the highest TPC value (135.2 ± 5.88 mg GAE/g), followed closely by the methanol extract (129.6 ± 5.48 mg GAE/g). The essential oil (122.2 ± 5.40 mg GAE/g) and *n*-butanol fraction (121.3 ± 5.37 mg GAE/g) showed nearly equal TPC values. The chloroform fraction displayed a moderate TPC level (101.9 ± 4.27 mg GAE/g), while the *n*-hexane fraction had the lowest TPC (35.2 ± 1.8 mg GAE/g). Statistical analysis confirmed significant differences in TPC values among the groups (*F*(5, 30) = 17.46, *p* < 0.001).

**Table 2:** Phenolic content, flavonoids, and TEAC of *A. rosea* extracts

Extract	TPC (mg GAE/g)	TFC (mg QE/g)	TEAC (µmol TE/g)
Essential Oil	122.2 ± 5.4	65.5 ± 3.3	349.1 ± 17.3
Ethyl Acetate	135.2 ± 5.9	60.1 ± 3.0	320.5 ± 16.0
Methanol	129.6 ± 5.5	50.2 ± 2.5	300.2 ± 15.0
*n*-Butanol	121.3 ± 5.37	40.6 ± 1.8	260.8 ± 13.0
Chloroform	101.9 ± 4.3	28.5 ± 1.5	120.3 ± 6.5
*n*-Hexane	35.2 ± 1.8	20.2 ± 1.2	75.0 ± 4.2

Regarding TFC (Table 2), the essential oil recorded the highest TFC (65.5 ± 3.3 mg QE/g), followed by the ethyl acetate (60.1 ± 3.0 mg QE/g). The *n*-hexane fraction showed the lowest TFC (20.2 ± 1.2 mg QE/g). A strong positive correlation (*r* = 0.85, *p* < 0.01) was observed between TPC values and the antioxidant activities of the extracts, indicating that phenolic compounds are major contributors to their antioxidant potential. The higher TPC and TFC in the ethyl acetate and methanol extracts, as well as the essential oil, corresponded well with their potent antioxidant activities observed in the various assays. These findings underscore the pivotal role of phenolic and flavonoid compounds in the antioxidant and DNA protective effects of *A. rosea* extracts, emphasising the importance of solvent selection for optimising the extraction of bioactive compounds.

### Antioxidant activities

The antioxidant capacities of *A. rosea* extracts and essential oil were assessed using three complementary in vitro assays: DPPH radical scavenging, FRAP, and OH· radical scavenging.

### DPPH radical scavenging activity

The DPPH radical scavenging activity was determined by measuring the IC_50_ (Table 3). All tested samples demonstrated significant antioxidant activity. The *n*-butanol fraction exhibited the highest antioxidant activity with the lowest IC_50_ value (18.5 ± 1.1 µg/mL). It was closely followed by the essential oil (IC_50_ = 19.8 ± 0.9 µg/mL) and the ethyl acetate (IC_50_ = 19.1 ± 1.0 µg/mL). In contrast, the *n*-hexane fraction displayed minimal DPPH radical scavenging activity, with an IC_50_ value exceeding 100 µg/mL. Statistical analysis (one-way ANOVA, *F*(6, 14) = 45.21, *p* < 0.001) confirmed significant differences in IC_50_ values among the tested samples. Post-hoc comparisons (Tukey’s HSD test) indicated that the *n*-butanol, essential oil, and ethyl acetate fractions were significantly more potent (*p* < 0.05) than the chloroform (IC_50_ = 38.1 ± 1.8 µg/mL) and *n*-hexane fractions. A strong positive correlation between extract concentration and inhibition percentages was observed for the *n*-butanol fraction (*r* = 0.94, *p* < 0.01) and essential oil (*r* = 0.92, *p* < 0.05), highlighting a dose-dependent radical-scavenging potential.

**Table 3:** Antioxidant (DPPH) and total reducing power of *A. rosea* extracts

Extract	DPPH Inhibition (%)	IC₅₀ (µg/mL)	FRAP (µmol Fe²⁺/g)	Relative Reducing Power (%)
Vitamin C	90.0 ± 0.1	15.6 ± 0.8 µM	350.2 ± 17.5	100.0
*n*-Butanol	90.0 ± 2.5	18.5 ± 1.1	270.5 ± 12.0	77.3
Essential Oil	82.0 ± 0.4	19.8 ± 0.9	300.1 ± 15.0	85.7
Ethyl Acetate	80.0 ± 3.0	19.1 ± 1.0	200.3 ± 10.5	57.2
Methanol	52.0 ± 3.3	45.3 ± 2.5	275.0 ± 13.0	78.5
Chloroform	48.0 ± 2.8	38.1 ± 1.8	150.0 ± 8.0	42.8
*n*-Hexane	22.0 ± 1.3	> 100	80.0 ± 5.5	22.8

### Ferric reducing antioxidant power (FRAP) and Trolox equivalent antioxidant capacity (TEAC)

**Fig. 2:**
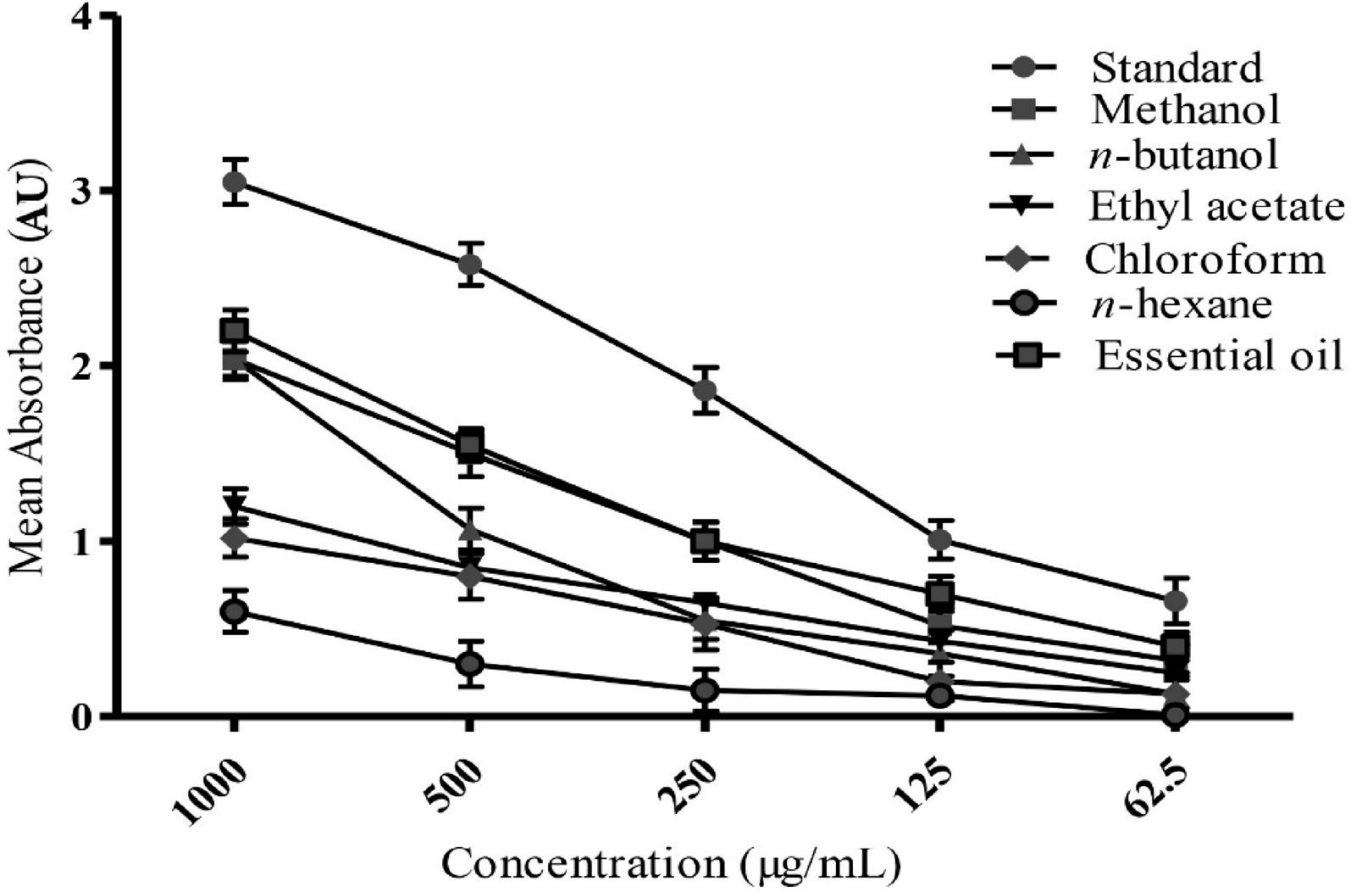
Antioxidant capacity of *A. rosea* extracts as determined by the FRAP assay. The figure shows absorbance values at 700 nm for methanol extract, *n*-hexane, chloroform, ethyl acetate, *n*-butanol fractions, and essential oil. Higher absorbance indicates greater reducing power. Ascorbic acid (AA) was used as the standard antioxidant

The FRAP assay measures the capacity of samples to reduce Fe³⁺ to Fe²⁺, with higher absorbance at 700 nm indicating stronger reducing power. As depicted in Fig. 2, the standard antioxidant, AA, demonstrated the highest absorbance (3.07 ± 0.06 AU). Among the *A. rosea* samples at 1000 µg/mL, the essential oil exhibited the highest absorbance (2.24 ± 0.05 AU), followed by the methanol extract (2.05 ± 0.04 AU) and the *n*-butanol fraction (2.04 ± 0.04 AU). The ethyl acetate showed moderate absorbance (1.17 ± 0.02 AU), while the chloroform (0.98 ± 0.02 AU) and *n*-hexane (0.55 ± 0.01 AU) fractions exhibited lower reducing capacities. Statistical analysis confirmed significant differences among the extracts (*F* = 94.12, *df* = 6, *p* < 0.001).

The quantitative FRAP values (µmol Fe²⁺/g), presented in Table 3, reinforce the reducing power of the extracts. The essential oil consistently exhibited the highest reducing capacity (300.1 ± 15.0 µmol Fe²⁺/g) among the *A. rosea* samples, followed by the methanol extract (275.0 ± 13.0 µmol Fe²⁺/g) and the *n*-butanol fraction (270.5 ± 12.0 µmol Fe²⁺/g). The ethyl acetate demonstrated a FRAP value of 200.3 ± 10.5 µmol Fe²⁺/g. The chloroform and *n*-hexane fractions displayed lower FRAP values (150.0 ± 8.0 and 80.0 ± 5.5 µmol Fe²⁺/g, respectively). When benchmarked against Vitamin C (350.2 ± 17.5 µmol Fe²⁺/g), the essential oil showed 85.7% relative reducing power, while the methanol extract and *n*-butanol fraction demonstrated 78.5% and 77.3%, respectively (Table 3).

TEAC values (Table 2) further supported these findings. The essential oil (349.1 ± 17.3 µmol TE/g) and ethyl acetate (320.5 ± 16.0 µmol TE/g) exhibited high TEAC values, consistent with their strong antioxidant potential. A strong positive correlation was observed between extract concentration and absorbance values (*r* = 0.87, *p* < 0.01), indicating a dose-dependent increase in reducing power. Correlation analysis (Table 4) also revealed strong relationships between FRAP values and both TPC (e.g., *r* = 0.94 for essential oil) and TFC (e.g., *r* = 0.92 for essential oil), indicating that these phytochemicals are primary contributors to the observed reducing power.

**Table 4:** Correlation coefficients between FRAP and phytochemical content

Extract	*r* (TPC)	*r* (TFC)
Essential Oil	0.94	0.92
Ethyl Acetate	0.91	0.88
Methanol	0.87	0.85
*n*-Butanol	0.85	0.84
Chloroform	0.76	0.72
*n*-Hexane	0.59	0.51

### Hydroxyl radical scavenging activity

OH^•^ radical scavenging activity, as presented in Table 5, provided complementary insights into the extracts’ ability to neutralise highly reactive OH^•^ radicals. The essential oil again demonstrated superior activity, with 78.4 ± 4.0% scavenging, followed by the ethyl acetate (71.5 ± 3.7%) and the methanol extract (62.3 ± 3.5%). The *n*-butanol fraction exhibited 58.2 ± 2.8% scavenging activity, while the chloroform (35.6 ± 2.2%) and *n*-hexane (22.1 ± 1.8%) fractions showed lower activity. These results generally align with the FRAP values, supporting the hypothesis that OH^•^ radical scavenging is closely tied to the overall antioxidant capacity of phenolic and flavonoid compounds.

**Table 5:** DNA protection, binding affinity, ROS inhibition, and hydroxyl radical scavenging

Extract	DNA Damage	Protection (%)	ROS Inhibition (%)	OH^•^ Scavenging (%)	DNA binding affinity (K_D_, µM)
Native DNA	0	–	–	–	–
H₂O₂ Control	90	–	–	–	–
Essential Oil	15	83	68	78.4 ± 4.0	1.2 ± 0.3
Methanol	20	78	62	62.3 ± 3.5	2.1 ± 0.5
Ethyl Acetate	25	72	65	71.5 ± 3.7	1.8 ± 0.4
*n*-Butanol	30	67	57	58.2 ± 2.8	3.3 ± 0.8
Chloroform	–	–	35.6 ± 2.2	35.6 ± 2.2	4.5 ± 1.0
*n*-Hexane	–	–	22.1 ± 1.8	22.1 ± 1.8	ND

The K_D_ further supports the protective effects. The essential oil exhibited the highest K_D_ (1.2 ± 0.3 µM, Table 5), indicating its strong interaction with DNA and ability to shield it from oxidative stress. Other fractions followed a trend generally consistent with their antioxidant capacities, with the chloroform fraction showing 4.5 ± 1.0 µM and *n*-hexane being “Not Determined” (ND).

### DNA protective effects

The protective potential of *A. rosea* extracts and essential oil against oxidative DNA damage was evaluated using the comet assay and the agarose gel-based DNA protection assay.

### Protection against oxidative DNA damage (comet Assay)

The comet assay demonstrated that pretreatment with the methanol extract and its fractions significantly mitigated H_2_O_2_-induced DNA damage. This was evidenced by reductions in the percentage of tail DNA, with values ranging from 12.4 ± 2.5% (*n*-butanol fraction) to 25.1 ± 3.1% (chloroform fraction). The essential oil exhibited a substantial protective effect, resulting in a tail DNA percentage of 15.6 ± 2.1%, which was comparable to the activity of the more potent *n*-butanol and ethyl acetate extracts. Consistent with its weaker antioxidant activity, the *n*-hexane fraction displayed a higher tail DNA percentage, indicating limited protective potential.

### DNA integrity, ROS inhibition (agarose gel assay) and DNA binding affinity

The DNA protection assay results (Tables 5 and 6) underscore the substantial protective effects of *A. rosea* extracts and essential oil against H_2_O_2_-induced oxidative damage on pBR322 DNA. These findings demonstrate the ability of *A. rosea* derivatives to inhibit ROS formation and maintain DNA integrity under oxidative stress.

In this assay, the native DNA (control, Lane 1, Table 6) remained intact and supercoiled, showing 0% DNA damage. Conversely, DNA treated with H_2_O_2_ alone (positive control, Lane 3, Table 6) exhibited extensive oxidative damage, with 90% DNA damage and predominance of the open circular form. Pretreatment with the essential oil (Lane 7, Table 6) demonstrated the strongest protection, reducing DNA damage to 15%, with a protective effect of 83% and ROS inhibition of 68%. The methanol extract (Lane 4, Table 6) reduced DNA damage to 20%, exhibiting a protective effect of 78% and ROS inhibition of 62%. The ethyl acetate (Lane 6, Table 6) also showed notable DNA safeguarding capabilities, reducing DNA damage to 25%, with a protective effect of 72% and ROS inhibition of 65%. The *n*-butanol fraction (Lane 5, Table 6) exhibited moderate efficacy, with DNA damage of 30%, a protective effect of 67%, and ROS inhibition of 57%. Notably, the chloroform fraction (Lane 8, Table 6) also showed a degree of protection, reducing DNA damage to 50%, with a 44% protective effect and 35.6% ROS inhibition. The *n*-hexane fraction (Lane 9, Table 6) provided minimal protection, showing 70% DNA damage, a 22% protective effect, and 22.1% ROS inhibition, consistent with its weakest antioxidant profile.

**Table 6:** DNA protection assay results

Sample	Lane number	DNA integrity	Description	DNA damage (%)	Protective effect (%)	Inhibition of ROS formation (%)
Control (Native DNA)	1	Intact, supercoiled	Native, undamaged pBR322 DNA	0	–	–
H_2_O_2_ (Positive Control)	3	Compromised, open circular	H_2_O_2_-induced damage	90	–	–
Essential Oil	7	Protected, supercoiled	Protective influence against H_2_O_2_-induced damage	15	83	68
Methanol Extract	4	Protected, supercoiled	Protective effect against H_2_O_2_-induced damage	20	78	62
Ethyl Acetate Fraction	6	Protected, supercoiled	Shielding effect against oxidative stress	25	72	65
*n*-Butanol Fraction	5	Protected, supercoiled	Shielding effect against oxidative stress	30	67	57
Chloroform fraction	8	Partially degraded	Mild protection with limited antioxidant response	50	44	35.6
*n*-Hexane	9	Severely degraded	Minimal protection, weak antioxidant response	70	22	22.1

### Protection against H_2_O_2_-induced haemolysis in RBCs

RBCs are crucial for oxygen transport, and their functional integrity is highly susceptible to oxidative damage, which can lead to impaired functionality and exacerbate disease progression [60]. Oxidative stress, particularly via lipid peroxidation, compromises RBC membrane integrity by altering fluidity and permeability [49]. This study investigated the protective efficacy of *A. rosea* extracts and essential oil against H_2_O_2_-induced haemolysis, aiming to demonstrate their potential in preserving RBC integrity.

In the experimental setup, RBCs exposed to H_2_O_2_ alone exhibited significant haemolysis, quantified at 83.21 ± 4.1%. For reference, Triton-X, a known haemolytic agent, served as a positive control and consistently induced 100% haemolysis.

**Fig. 3:**
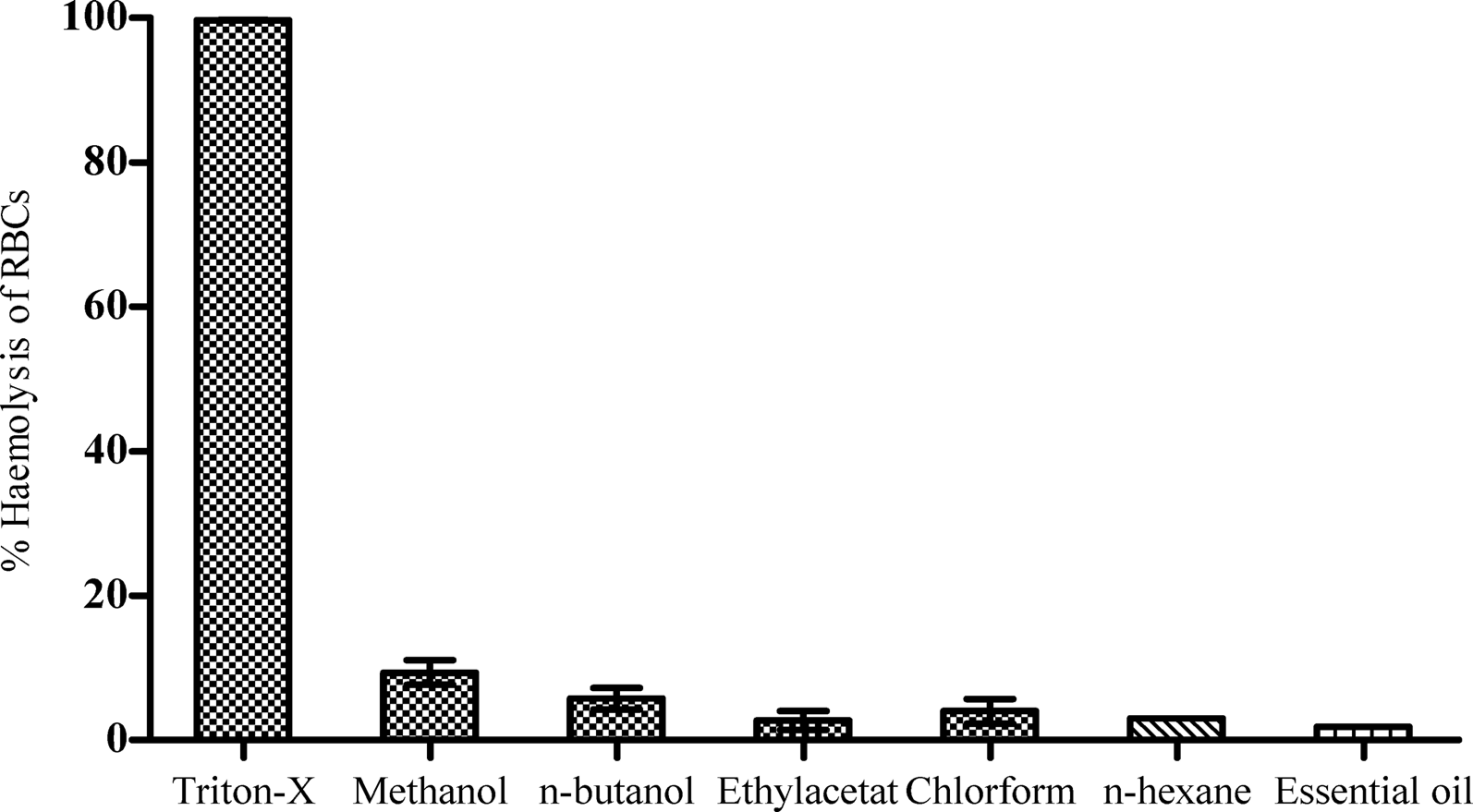
In-vitro haemolytic activity of *A. rosea* extracts

The protective efficacy of *A. rosea* extracts and fractions against H_2_O_2_-induced haemolysis in RBCs was evaluated (Fig. 3). Complete haemolysis (100%) was observed with Triton-X. In contrast, the essential oil demonstrated the most potent protective effect, limiting haemolysis to a remarkably low 1.62 ± 0.3%. Among the solvent extracts, ethyl acetate exhibited the lowest haemolysis at 3.21%, followed by *n*-hexane (3.67%), chloroform (4.59%), and *n*-butanol (6.88%). The methanol extract displayed a dose-dependent protective effect, with haemolysis percentages ranging from 1.88 ± 0.7% to 9.36 ± 3.5%.

These compelling findings align well with the overall antioxidant capacities previously observed for *A. rosea* extracts in assays such as DPPH radical scavenging, total reducing power (Table 3), and ROS inhibition (Table 5). The high protective efficacy against H_2_O_2_-induced haemolysis is likely attributable to the rich profile of bioactive compounds, including flavonoids, phenolic acids, and terpenes, which are well-documented for their radical-scavenging and redox-modulating properties [49]. By neutralising ROS and maintaining redox balance within RBCs, these compounds help to preserve cellular and membrane integrity. The observed dose-dependent responses further underscore the therapeutic potential of these extracts in mitigating oxidative stress. Furthermore, the low inherent haemolytic activity of the extracts themselves supports their overall safety profile and suggests that the observed protective effects are due to their antioxidant properties rather than any membrane-damaging effects.

In summary, this study unequivocally demonstrates the significant protective effects of *A. rosea* extracts against H_2_O_2_-induced haemolysis in RBCs, highlighting their promise as valuable natural agents in the management of oxidative stress-related disorders.

### Cytotoxicity profile of *A. rosea* extracts and essential oil

The cytotoxic potential of *A. rosea* extracts and essential oil was evaluated via a modified haemolytic assay using human erythrocytes. As expected, the positive control (0.1% Triton X-100) induced complete RBC lysis (100.00 ± 0.00%), while the negative control (PBS) exhibited negligible lysis (0.56 ± 0.05%), confirming baseline membrane integrity (Table 7).

At a concentration of 1 mg/mL, all tested *A. rosea* samples demonstrated very low haemolytic activity, with values significantly lower than the Triton X-100 control and comparable to the PBS negative control, indicating minimal membrane-damaging potential (Table 7). The specific haemolysis percentages for each sample are presented in Table 7. These findings collectively affirm the favourable cytotoxicity profile of *A. rosea* extracts and essential oil on human erythrocytes under the experimental conditions.

**Table 7:** Haemolytic activity of *A. rosea* extracts and essential oil at 1 mg/ml

Sample	Haemolysis (%)
PBS (Negative Control)	0.56 ± 0.05
Triton X-100 (Positive Control)	100.00 ± 0.00
Methanol extract	2.98 ± 0.25
*n*-Butanol Fraction	3.23 ± 0.28
Ethyl acetate fraction	2.65 ± 0.20
Essential Oil	2.42 ± 0.22
Chloroform fraction	4.18 ± 0.30
*n*-Hexane fraction	5.36 ± 0.35

## Discussion

The present investigation provides compelling evidence of the potent antioxidant and cytoprotective capacities of *A. rosea* extracts and essential oil, thereby substantiating its ethnobotanical use and rigorously validating its bioactivity through a suite of mechanistic assays. In a world increasingly challenged by chronic diseases rooted in oxidative stress, the discovery and scientific validation of natural agents like *A. rosea* are paramount. Antioxidant defence is a central pillar in combating oxidative stress, a pathological hallmark implicated in the aetiology of numerous chronic diseases, including cancer, neurodegeneration, cardiovascular disease, and metabolic syndromes [49]. Our findings unequivocally demonstrate that *A. rosea* extracts possess pronounced free radical scavenging capabilities, effectively reducing oxidative stress markers and preserving biomolecular integrity in vitro, offering a promising avenue for therapeutic development.

The observed antioxidant potential, particularly evident in the *n*-butanol, ethyl acetate, and methanol fractions, suggests that *A. rosea* is a rich and diverse source of compounds capable of neutralising harmful free radicals. While the IC₅₀ values in the DPPH assay for these fractions were comparatively higher than that of the standard AA, the substantial FRAP of the essential oil, methanol, and *n*-butanol fractions highlights their significant contribution to the overall antioxidant capacity (Table 3). This discrepancy between DPPH and FRAP assays can be interpreted as a reflection of the varied mechanisms of action of the phytochemicals present: DPPH primarily assesses radical scavenging via hydrogen (H) atom transfer, while FRAP measures electron-donating capacity [43, 53]. The robust activity across both assays underscores a comprehensive antioxidant profile, indicating that *A. rosea* compounds can engage in multiple redox reactions. This potent activity is consistent with the well-documented properties of polyphenolic and flavonoid compounds, which are abundant in *A. rosea* and known for their H-donating and metal-chelating abilities [61, 62]. These compounds play a crucial role in terminating radical chain reactions and reducing the risk of oxidative biomolecular damage [63]. Comparative studies on other medicinal plants, such as *Hypericum hyssopifolium L.* [62] and various *Origanum* species [35], also attribute their significant antioxidant capacities to similar phenolic and flavonoid profiles, reinforcing the general principle that these compound classes are key players in plant-derived antioxidant benefits. The multi-faceted antioxidant capacity observed across different assays underscores the comprehensive protective effects of *A. rosea* against various forms of oxidative challenge, suggesting its potential efficacy against a broad spectrum of ROS.

Further mechanistic insights were gained from the GC-MS profiling of *A. rosea* essential oil, which revealed the presence of key bioactive components such as sesquiterpenes (e.g., *β*-caryophyllene) and oxygenated monoterpenes (e.g., carvacrol, linalool). These compounds are widely recognised for their established roles in modulating oxidative pathways and cellular defence mechanisms [64, 65]. For instance, *β*-caryophyllene is known to activate the nuclear factor erythroid 2-related factor 2 (Nrf2) pathway, a critical regulator of endogenous antioxidant response [66]. Nrf2 activation is a sophisticated cellular strategy, leading to the upregulation of genes encoding antioxidant enzymes (e.g., superoxide dismutase, catalase, glutathione peroxidase) and phase II detoxifying enzymes, thereby enhancing the cell’s intrinsic capacity to combat oxidative stress and remove harmful electrophiles [67]. This endogenous defence mechanism provides a more sustainable and robust protection compared to direct antioxidant scavenging alone. Similarly, carvacrol exhibits potent radical-scavenging and membrane-stabilising effects [68]. Its ability to integrate into lipid bilayers can alter membrane fluidity, thereby protecting against lipid peroxidation, a critical event in oxidative cellular damage. The presence of such compounds provides a clear chemical basis for the observed biological activities, suggesting that the diverse phytochemical profile of *A. rosea* contributes synergistically to its overall antioxidant and cytoprotective properties. These findings align with other studies investigating the antioxidant potential of plant essential oils and extracts rich in similar terpenoids and phenolics, such as *Myrtus communis L.* [65] and *Prunus armeniaca* [69]. From a plant biological perspective [14], the production of these diverse antioxidant compounds by *A. rosea* likely serves as an adaptive mechanism, enabling the plant to cope with environmental stressors, including UV radiation, drought, and pathogen attacks, which inherently induce oxidative stress within plant tissues. This evolutionary strategy for self-preservation translates into therapeutic potential for human health.

The cytoprotective mechanisms of *A. rosea* extracts were further elucidated through the DNA protection (comet) assay and the haemolysis assay. In the comet assay, *A. rosea* extracts significantly ameliorated DNA damage induced by oxidative stress, characterised by a reduction in tail length and tail moment in H_2_O_2_-treated lymphocytes. This protective effect is likely due to the extracts’ ability to inhibit the Fenton reaction by chelating Fe²⁺ ions, thereby preventing the generation of highly damaging OH^•^ radicals [70]. The high phenolic and flavonoid content, particularly in the more active fractions, strongly supports this mechanism, as these compounds are well-known metal chelators [27]. This mechanism has been extensively documented for other polyphenol-rich plant extracts, such as those from *Cinnamomum zeylanicum* [57] and various fruit extracts [71], reinforcing the plausibility and significance of this protective pathway in *A. rosea*. For the plant itself, maintaining DNA integrity is crucial for survival, growth, and reproduction, especially in environments prone to oxidative stress from various abiotic and biotic factors [72].

Complementarily, the haemolysis assay demonstrated a clear protective effect against H_2_O_2_-induced erythrocyte lysis. RBCs are particularly vulnerable to oxidative damage due to their high polyunsaturated fatty acid content and reliance on intrinsic antioxidant enzymes [23, 24]. The observed protection indicates that *A. rosea* extracts enhance membrane stability by inhibiting lipid peroxidation, a key event leading to increased membrane permeability and cell lysis [73]. The remarkably low haemolysis percentages observed for the methanol extract and essential oil further support their significant membrane-stabilising capabilities. This protective capacity of *A. rosea* against RBC oxidative damage is consistent with other studies on natural antioxidants, such as hesperidin [48] and extracts from *Carissa carandas* [54], which also demonstrate membrane-stabilising effects. The preservation of erythrocyte integrity is of profound biological implication, as compromised RBCs can lead to impaired oxygen transport, increased systemic oxidative stress, and contribute to various pathological conditions [2, 20, 48].

The protective effects observed across both DNA and RBC models are not isolated phenomena [48] but appear to be interconnected through a broader redox-modulatory mechanism. The strong correlation between the observed antioxidant potency (e.g., IC₅₀ and FRAP values) and the protective outcomes in cellular assays (comet and haemolysis) suggests a direct causative link. Specifically, the phenolic-rich fractions effectively reduce the ROS burden, which in turn prevents macromolecular oxidation and cellular dysfunction [74]. This relationship underscores the therapeutic potential of *A. rosea* not just as a general antioxidant supplement but as a promising candidate for cytoprotective interventions in conditions characterised by redox imbalance. The multi-faceted protective capacity highlights the potential synergistic or complementary interplay among the diverse phytochemical classes identified, including both the prominent terpenoids in the essential oil and the abundant phenolic/flavonoid compounds in the extracts, in conferring robust cellular and molecular defence. This synergy is a common feature in phytomedicine, where the combined action of multiple compounds often surpasses the effect of isolated constituents. Importantly, the low inherent haemolytic activity of the extracts themselves further supports their safety profile, suggesting that the observed protective effects are primarily attributable to their antioxidant properties rather than any membrane-damaging effects [25].

Unlike many natural product studies that demonstrate only general antioxidant properties, this study provides mechanistic specificity and quantitative validation, significantly strengthening its translational relevance. The comprehensive approach, employing a broad spectrum of assays from free radical scavenging to cellular protection, offers a multidimensional understanding of *A. rosea*’s biological impact. These findings not only reinforce the rationale for its traditional medicinal use but also pave the way for future pharmacological development and potential therapeutic applications in oxidative stress-related disorders, positioning *A. rosea* as a valuable lead for novel antioxidant-based therapies.

### Delimitations

This study focused exclusively on the evaluation of *A. rosea*, and the findings may not be generalisable to other plant species. The investigation was limited to the assessment of the plant’s antioxidant potential and protective effects, and did not explore in deep its potential therapeutic applications or toxicity profiles.

### Limitations

This comprehensive investigation of *A. rosea*’s phytochemical composition and biological activities is not without its limitations. A primary constraint lies in the extraction and fractionation processes, which may have inadvertently altered the composition and bioactivity of the resulting extracts and fractions [69]. This underscores the importance of optimising these processes to ensure the accuracy and reliability of the results. Another limitation stems from the reliance on in vitro assays, which may not accurately translate to in vivo effects. While these assays provide valuable insights into the biological activities of the plant extracts and fractions, they may not fully capture the complex interactions that occur within living organisms [75, 76]. Therefore, future studies should consider incorporating in vivo experiments to validate the findings and provide a more comprehensive understanding of the plant’s potential.

Furthermore, the solubility and stability of the bioactive compounds in various solvents employed may have influenced the results. The choice of solvent can significantly impact the extraction efficiency and stability of the bioactive compounds, which in turn may affect their biological activities. To mitigate this limitation, future studies should consider employing a range of solvents and optimising the extraction conditions to ensure the maximum recovery and stability of the bioactive compounds. Consequently, while this study provides valuable insights into the antioxidant potential and protective effects of *A. rosea*, it is essential to acknowledge the limitations and consider them in the design and interpretation of future studies. By addressing these limitations, researchers can further elucidate the biological activities of this plant and unlock its full potential for therapeutic applications.

## Recommendation and future directions

In light of the demonstrated antioxidant efficacy and protective effects of *A. rosea* extracts and essential oil against oxidative damage to DNA and RBCs, we recommend their continued investigation as promising candidates for therapeutic development targeting oxidative stress-related pathologies, including cancer, neurodegenerative diseases, and cardiovascular disorders.

To advance the translational potential of *A. rosea*, future studies should prioritise the following areas:

i. Mechanistic Elucidation: Detailed molecular investigations are required to elucidate the specific biochemical pathways and cellular targets modulated by *A. rosea* phytoconstituents. This includes exploring the modulation of endogenous antioxidant defences (e.g., Nrf2/antioxidant response element (ARE) pathway), inhibition of ROS-generating enzymes, and DNA repair mechanisms.
ii. Bioactive Compound Isolation: While the present study establishes a clear link between antioxidant activity and extract potency, isolation and structural characterisation of the key active constituents (e.g., terpenes, flavonoids, phenolic acids) through bioassay-guided fractionation will be critical for standardisation and drug development.
iii. Synergistic Interactions: Investigating the synergistic effects among individual compounds within the extracts may uncover enhanced bioactivity, reduce required dosages, and minimise potential cytotoxicity. This approach is particularly relevant for multi-target diseases involving complex redox imbalances.
iv. Pharmacological and Toxicological Profiling: Rigorous in vivo studies are needed to evaluate the pharmacokinetics, bioavailability, therapeutic index, and safety profile of *A. rosea*-derived products. These data are essential for validating their suitability for clinical or nutraceutical application.
v. Applied Potential in Industry: Given their antioxidative stability and bioactivity, *A. rosea* extracts may hold value not only in pharmaceuticals but also in the cosmetic and food industries as natural preservatives or functional ingredients. Controlled formulation and efficacy testing in these matrices should be pursued.

Overall, these directions will facilitate the rational development of *A. rosea*-based products and establish a scientific framework for their integration into modern therapeutic and preventive strategies against oxidative stress-mediated disorders.

## Conclusion

This study definitively validates the potent antioxidant and cytoprotective properties of *A. rosea* extracts and essential oil. Our integrated approach, combining phytochemical profiling with diverse in vitro assays, demonstrated its broad-spectrum antioxidant capacity. Key findings include significant protection against H_2_O_2_-induced DNA damage and erythrocyte haemolysis, attributed to its rich profile of polyphenolic, flavonoid, and terpenoid compounds. With favourable cytotoxicity, this research scientifically repositions *A. rosea* as a promising natural agent for therapeutic and industrial applications in managing oxidative stress-related pathologies. Vitally, all tested extracts and the essential oil exhibited minimal haemolytic activity, reinforcing their low cytotoxicity and supporting their safety for prospective pharmaceutical and therapeutic applications.

## Electronic supplementary material

Below is the link to the electronic supplementary material.

Supplementary Material 1

Supplementary Material 2

## Data Availability

The datasets used and/or analysed during the current study are available from the corresponding author upon reasonable request.

## Abbreviations

AA: Ascorbic acid
ABTS·+: 2,2′-azino-bis(3-ethylbenzothiazoline-6-sulfonic acid
ANOVA: One-way analysis of variance
ARE: Antioxidant response element
*A*. *rosea*: Aitchisonia rosea
CLs: Confidence limits
CV: Coefficient of variation
DMRT: Duncan’s multiple range test
DPPH: 2,2-diphenyl-1-picrylhydrazyl
FRAP: Ferric reducing antioxidant power assay
GA: Gallic acid
GC-MS: Gas chromatography-mass spectrometry
HCA: Hierarchical Cluster Analysis
IC50: Half-maximal inhibitory concentration
ICH: International Conference on Harmonisation
KD: Equilibrium dissociation constant
LOD: Limit of detection
LOQ: Limit of quantitation
NIST: National Institute of Standards and Technology
Nrf2: Nuclear factor erythroid 2-related factor 2
OT: Oven temperature
PBS: Phosphate-buffered saline
PCA: Principal Component Analysis
PMA: Phosphomolybdic acid
PTA: Phosphotungstic acid
R^2^: Coefficient of determination
RBC: Red blood cell
RI: Retention indices
ROS: Reactive oxygen species
RT: Retention time
SEM: Standard error of the mean
SPSS: Statistical Package for the Social Sciences
TBARS: Thiobarbituric acid reactive substances
TBE: Tris-Borate-EDTA
TEAC: Trolox Equivalent Antioxidant Capacity
TFC: Total flavonoid content
TPC: Total phenolic content
Trolox: 6-hydroxy-2,5,7,8-tetramethylchroman-2-carboxylic acid
