# Supplementary figures and images for: Harnessing the antioxidant and cytoprotective power of Aitchisonia rosea: phytochemical insights and mechanistic validation
Source: BMC Plant Biol. 2025 Aug 22;25:1116. doi: 10.1186/s12870-025-07084-7 (PMC12372252; doi:10.1186/s12870-025-07084-7)

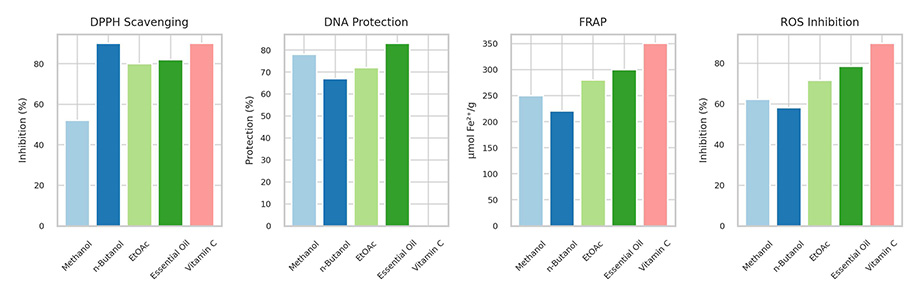

Supplement: Supplementary file 1 — Supplementary Material 1 [file 12870_2025_7084_MOESM1_ESM.jpg]
