# Supplementary material for: Harnessing the antioxidant and cytoprotective power of Aitchisonia rosea: phytochemical insights and mechanistic validation
Source: BMC Plant Biol. 2025 Aug 22;25:1116. doi: 10.1186/s12870-025-07084-7 (PMC12372252; doi:10.1186/s12870-025-07084-7)
Supplement: Supplementary file 2 — Supplementary Material 2 [file 12870_2025_7084_MOESM2_ESM.pdf]

Supplementary Material

Harnessing the Antioxidant and Cytoprotective Power of *Aitchisonia rosea*: Phytochemical Insights and Mechanistic Validation

Loai Aljerf[1,2**], Abdullah H. Maad[3], Shahid Rasool[4] and Muaaz Alajlani[1*]

[1] *Faculty of Pharmacy, Al-Sham Private University, Damascus 5910011, Syrian Arab Republic*

[2] *Key Laboratory of Organic Industries, Department of Chemistry, Faculty of Sciences, Damascus University, Damascus, Syrian Arab Republic*

[3] *Department of Pharmaceutics, Collage of Pharmacy, University of Al-Ameed, Karbala City, Iraq*

[4] *College of Pharmacy, University of Sargodha, Sargodha-44100, Pakistan*

[*]Correspondence:
Muaaz Alajlani
Muaaz.alajlani.foph@aspu.edu.sy

Loai Aljerf
loai_aljerf.foph@aspu.edu.sy, loai789.aljerf@damascusuniversity.edu.sy

1. The Power of Medicinal Plants in Modern Healthcare

Medicinal plants have been a cornerstone of traditional medicine for centuries, and their significance cannot be overstated in today's world. The importance of these plants lies in their ability to provide natural remedies for various health issues, often with fewer side effects compared to synthetic medicines. Among the plethora of medicinal plants, *Aitchisonia albens*, commonly known as *Aitchisonia rosea* (*A. rosea*) (Scheme 1) stands out for its remarkable therapeutic properties. *A. rosea*, a species of flowering plant native to the Himalayan region, has been used in traditional medicine for centuries to treat various ailments, including fever, rheumatism, and digestive issues. The plant contains a rich array of bioactive compounds, including flavonoids, alkaloids, and phenolic acids, which have been shown to possess antioxidant, anti-inflammatory, and antimicrobial properties (in this study). These properties make *A. rosea* potentially an attractive candidate for the treatment of oxidative stress-related disorders, which are a leading cause of chronic diseases such as cancer, cardiovascular disease, and neurodegenerative disorders. One of the primary mechanisms by which *A. rosea* exerts its therapeutic effects is by enhancing antioxidant defences. Oxidative stress occurs when the body's natural antioxidant defences are overwhelmed by reactive oxygen species (ROS), leading to cellular damage and tissue injury. *A. rosea*'s antioxidant properties help to neutralise ROS, thereby protecting cells from damage and promoting overall health.

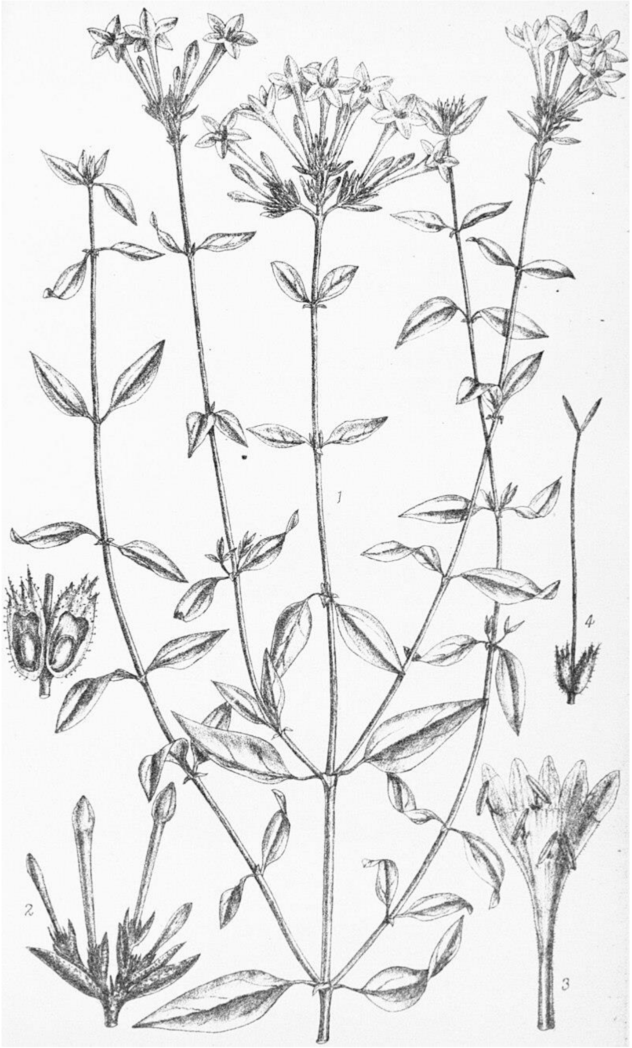

Scheme 1. Botanical illustration of *Aitchisonia rosea* from the protologue of the genus and species

Another medicinal plant that has gained significant attention in recent years is *Vigna unguiculata*, also known as cowpea (Sardar et al., 2024). This legume is rich in protein, fibre, and various micronutrients, making it an excellent addition to a healthy diet. *Vigna unguiculata* has been shown to possess antioxidant, anti-inflammatory, and antimutagenic properties, which have been linked to its ability to protect against chronic diseases such as cancer, diabetes, and cardiovascular disease.

Cannabis oil, derived from the cannabis plant, is another medicinal plant that has been gaining recognition for its therapeutic potential. The oil contains a range of bioactive compounds, including cannabinoids, flavonoids, and terpenes, which have been shown to possess anti-inflammatory, antioxidant, and analgesic properties. Cannabis oil has been used to treat a range of health issues, including pain management, anxiety, and sleep disorders. In fact, a recent study published in Biomed Pharmacother demonstrated the efficacy of broad-spectrum cannabis oil in ameliorating reserpine-induced fibromyalgia in mice, highlighting its potential as a therapeutic agent for managing chronic pain (Ferrarini et al., 2022).

One of the most significant benefits of medicinal plants like *A. rosea*, *Vigna unguiculata*, and cannabis oil is their ability to protect DNA from damage. Oxidative stress and inflammation can lead to DNA damage, which can contribute to the development of chronic diseases. The antioxidant properties of these plants help to neutralise ROS, thereby protecting DNA from damage and promoting genomic stability. In addition to their individual benefits, medicinal plants like *A. rosea*, *Vigna unguiculata*, and cannabis oil may also have synergistic effects when used in combination. For example, the antioxidant properties of *A. rosea* may enhance the anti-inflammatory effects of cannabis oil, leading to improved therapeutic outcomes. Similarly, the protein-rich content of *Vigna unguiculata* may enhance the bioavailability of the bioactive compounds, leading to improved therapeutic effects. So, medicinal plants like *A. rosea*, *Vigna unguiculata*, and cannabis oil are a treasure trove of natural remedies that hold immense potential for promoting human health. Their antioxidant, anti-inflammatory, and antimicrobial properties make them attractive candidates for the treatment of oxidative stress-related disorders, and their ability to protect DNA from damage is a significant advantage in the prevention of chronic diseases. As the world continues to grapple with the challenges of healthcare, it is essential that we continue to explore

the therapeutic potential of medicinal plants and integrate them into our healthcare systems. Hence, the importance of medicinal plants in modern healthcare cannot be overstated. Thus, it is essential that we explore alternative approaches to healthcare that prioritise prevention and natural remedies. Medicinal plants like *A. rosea*, *Vigna unguiculata*, and cannabis oil offer a safe, effective, and sustainable approach to healthcare that can be integrated into our existing healthcare systems. Furthermore, the incorporation of medicinal plants into healthcare systems can also help to reduce the economic burden of chronic diseases, making healthcare more accessible and affordable for individuals around the world. By embracing the therapeutic potential of medicinal plants, we can create a more holistic and sustainable approach to healthcare that prioritises the well-being of individuals and the planet. Furthermore, the use of medicinal plants can also help to reduce the burden on our healthcare systems. By promoting preventive healthcare and natural remedies, we can reduce the incidence of chronic diseases, which are a significant drain on our healthcare resources. This approach can also help to reduce the economic burden of healthcare, making it more accessible and affordable for individuals and communities. In addition, the use of medicinal plants can also help to promote sustainable agriculture and conservation. Many medicinal plants are grown using sustainable agricultural practices, which can help to promote environmental sustainability and conserve biodiversity.

In terms of future research directions, there are several areas that warrant further exploration. One area is the development of standardised extracts and formulations of medicinal plants, which can help to ensure consistency and efficacy in their therapeutic applications. Another area is the investigation of the synergistic effects of medicinal plants when used in combination, which can help to enhance their therapeutic potential. In conclusion, medicinal plants like *A. rosea*, *Vigna unguiculata*, and cannabis oil are a valuable resource that holds immense potential for promoting

human health and well-being. Their antioxidant, anti-inflammatory, and antimicrobial properties make them attractive candidates for the treatment of oxidative stress-related disorders, and their ability to protect DNA from damage is a significant advantage in the prevention of chronic diseases. As we move forward, it is essential that we continue to explore the therapeutic potential of medicinal plants, develop standardised extracts and formulations, and investigate their synergistic effects when used in combination. By doing so, we can unlock the full potential of medicinal plants and promote a healthier, more sustainable future for all.

2. Calibration Curve Construction and TPC Calculation

A calibration curve for gallic acid (GA) was constructed using linear dose-response regression analysis on an Excel spreadsheet, with concentrations of 10, 20, 40, 80, 100, and 120 µg/mL (Fig. S1). The TPC was expressed as milligrams of GA equivalents (GAE) per gram of sample, with a CL of 95% and a precision of ± 2%. This method has been demonstrated to be reliable and reproducible, with a CV of less than 5% (Singleton et al., 1999).

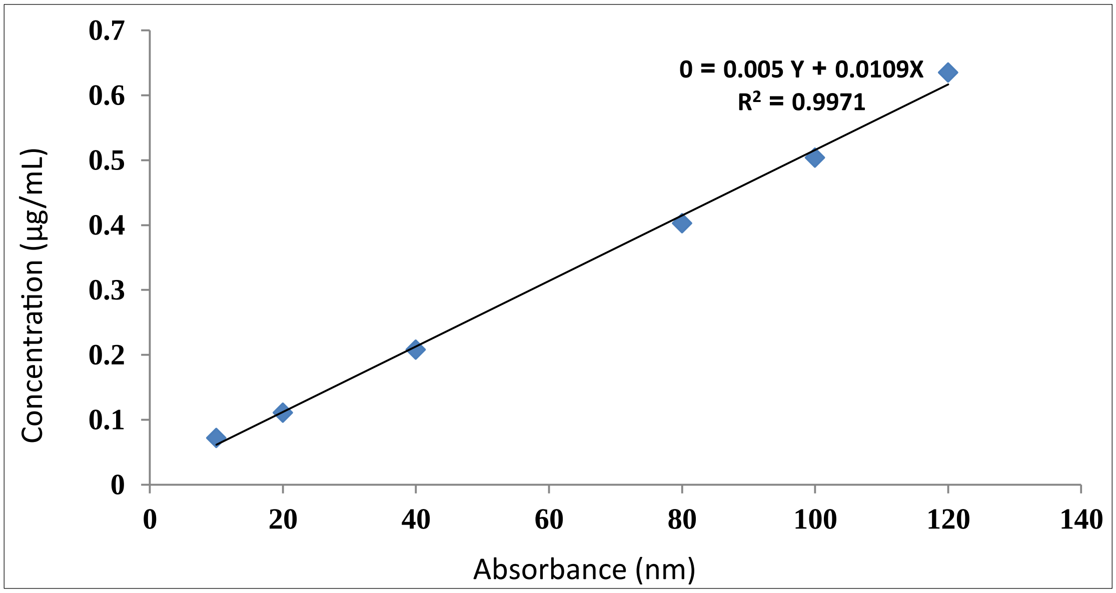

Fig. S1. Calibration curve for gallic acid: a linear regression analysis.

3. Chemical Composition and Bioactive Compounds of *A. rosea* Essential Oil

An in-depth analysis of *A. rosea* essential oil reveals a rich and diverse chemical composition, as characterised by GC-MS analysis (Table 1, Fig. S2). This analysis identified 27 compounds, which can be classified into monoterpene hydrocarbons, oxygenated monoterpenes, sesquiterpene hydrocarbons, oxygenated sesquiterpenes, and fatty acid methyl esters. The total yield of the essential oil was 0.27% (w/w dry plant material), with 99.9% of its chemical composition identified.

The GC-MS chromatogram (Fig. S2) highlights 27 distinct peaks, corresponding to compounds such as germacrene, carvacrol, *β*-caryophyllene, linalool, and octadecanoic acid methyl ester. These compounds are recognised for their significant bioactivities, which contribute to the oil's functional properties.

Chemical Composition

1. Monoterpene Hydrocarbons (9.42%)

   This group includes *α*-pinene (0.79%), camphene (2.48%), *β*-pinene (1.27%), myrcene (0.73%), cymene (1.50%), and limonene (0.65%). These compounds are associated with antimicrobial, anti-inflammatory, and antioxidant properties.

2. Oxygenated Monoterpenes (7.41%)

   Oxygenated monoterpenes such as linalool (2.67%), carveol (0.80%), verbenone (0.98%),

and thymol (2.96%) contribute to the oil's antioxidant and antimicrobial effects, with specific emphasis on their roles in mitigating oxidative stress and microbial proliferation.

3. Sesquiterpene Hydrocarbons (36.19%)
   The largest category, sesquiterpene hydrocarbons, includes germacrene (18.43%), *β*-caryophyllene (5.89%), valencene (2.78%), cadinene (3.02%), spathulenol (3.66%), and *α*-copaene (1.72%). These compounds exhibit potent anti-inflammatory, antioxidant, and antimicrobial properties.

4. Oxygenated Sesquiterpenes (5.18%)
   This category includes *α*-muurolol (3.75%) and *α*-cadinol (1.43%), which contribute to the essential oil's antioxidant and antimicrobial effects.

5. Fatty Acid Methyl Esters (16.25%)
   Fatty acid methyl esters such as hexadecanoic acid methyl ester (1.82%) and octadecanoic acid methyl ester (14.43%) enhance the bioactivity of the essential oil, particularly its antioxidant and antimicrobial potential.

Functional Implications and Synergistic Interactions

The bioactivity of *A. rosea* essential oil is underpinned by the interplay of its chemical constituents. Compounds like germacrene, carvacrol, and *β*-caryophyllene, identified as high-abundance compounds (Cluster 1 in the Hierarchical Cluster Analysis (HCA)), play a dominant role in shaping the oil's antioxidant and antimicrobial effects. Similarly, moderate-abundance compounds such as linalool and carveol (Cluster 2) may contribute sedative effects, as highlighted by their grouping in Principal Component Analysis (PCA) analysis.

The strong positive correlation between *β*-caryophyllene and germacrene ($r = 0.83$) suggests potential shared biosynthetic pathways, while the moderate correlation between linalool and carveol ($r = 0.56$) hints at functional synergy. These findings underscore the importance of understanding both individual compound activity and synergistic interactions.

Broader Applications

The intricate chemical profile of *A. rosea* essential oil positions it as a promising candidate for applications in the pharmaceutical, cosmetic, and food industries. Further studies are recommended to:

- Investigate individual compound activities: For deeper insights into their therapeutic roles.
- Examine synergistic effects: To understand the enhanced bioactivities resulting from compound interactions.
- Explore functional applications: Including drug formulation, food preservation, and cosmetic development.

The detailed insights from GC-MS analysis (Fig. S2 and Table 1) provide a strong foundation for leveraging the diverse bioactivities of *A. rosea* essential oil in various fields, emphasising its multifaceted potential.

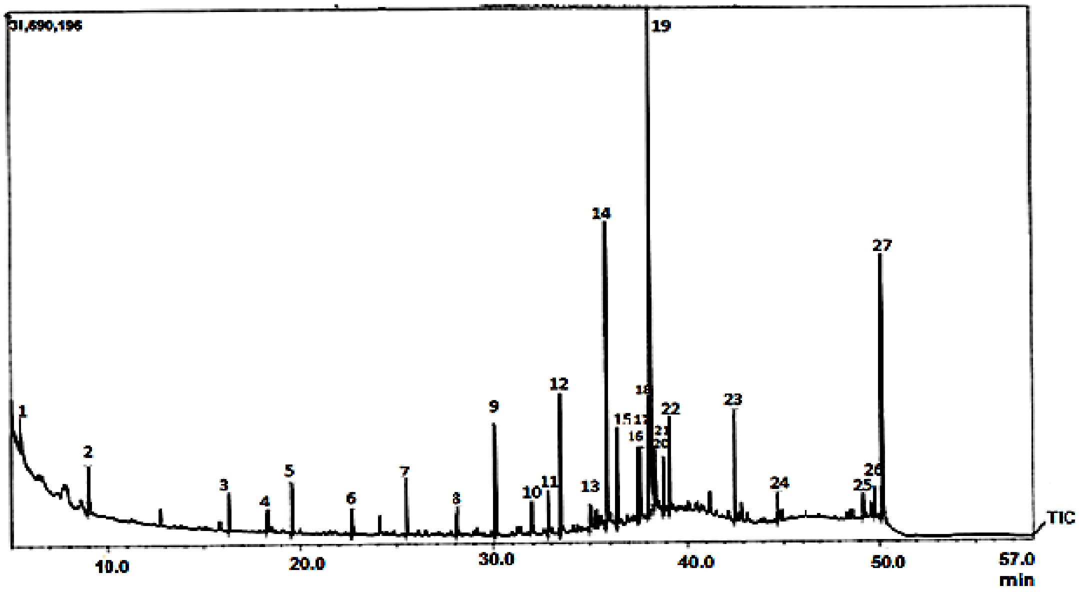

Fig. S2. GC-MS chromatogram of *A. rosea* essential oil, exhibiting 27 distinct peaks, which, from left to right, correspond to the following compounds: Limonene, Myrcene, Undecanone, *β*-Phellandrene, *α*-Pinene, Carveol, Verbenone, *β*-Pinene, *α*-Cadinol, Cymene, *α*-Copaene, Cineole, Hexadecanoic acid methyl ester, Eugenol, Eicosane, Camphene, *β*-Elemene, Linalool, Valencene, Thymol, Cadinene, Spathulenol, *α*-Muurolol, *β*-Caryophyllene, Carvacrol, Octadecanoic acid methyl ester, and Germacrene, demonstrating the comprehensive chemical composition of the essential oil.

4. DNA Protection Assay Results

The DNA protection assay was conducted to evaluate the ability of *A. rosea* methanolic extract, ethyl acetate fraction, *n*-butanol fraction, and essential oil to protect DNA from oxidative damage. The assay utilised pBR322 plasmid DNA exposed to hydrogen peroxide (H_2O_2) at a concentration

of 1000 µg/mL for each test sample, selected to assess their maximum protective efficacy. The results of this assay, as visualised through gel electrophoresis, are presented in Fig. S3.

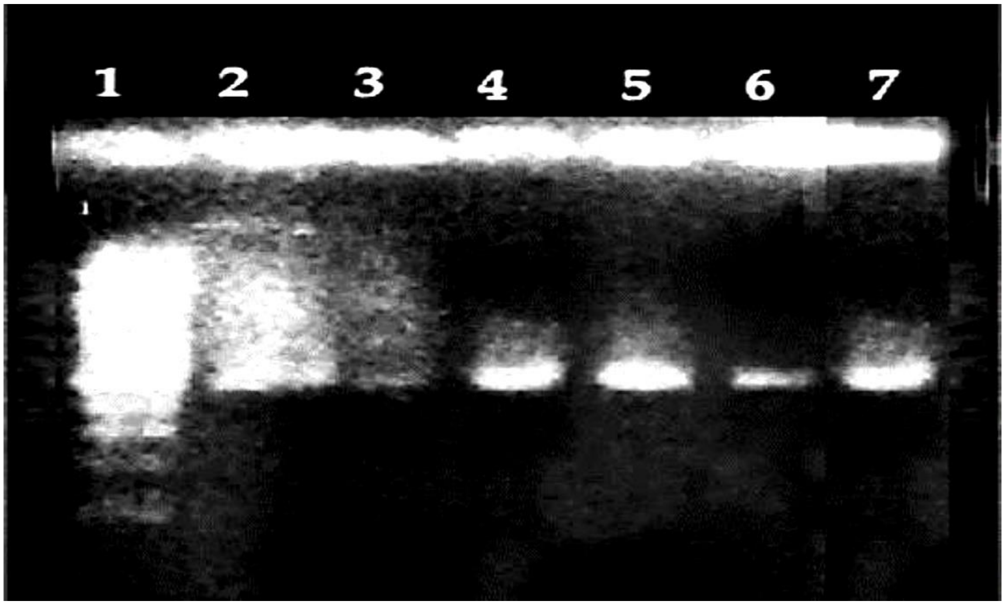

Fig. S3. Electropherogram showing DNA protection effect by *A. rosea* extract, various organic fractions and essential oil with H_2O_2 induced oxidative damage on pBR322 DNA. (Lane 1 = Plasmid pBR322 DNA without treatment (Supercoiled); Lane 2 = Plasmid pBR322 DNA and dye; Lane 3 = Plasmid pBR322 DNA treated with H_2O_2 (open circular or damaged); Lane 4 = Plasmid pBR322 DNA treated with methanol extract + H_2O_2; Lane 5 = Plasmid pBR322 DNA treated with *n*-butanol fraction + H_2O_2; Lane 6 = Plasmid pBR322 DNA treated with ethyl acetate fraction + H_2O_2; Lane 7 = Plasmid pBR322 DNA treated with essential oil + H_2O_2).

As depicted in Fig. S3, Lane 1 represents the control, showing the native supercoiled form of pBR322 DNA, which remained intact and undamaged. In contrast, Lane 3, containing pBR322

DNA treated solely with H_2O_2, demonstrates extensive oxidative damage, indicated by the predominance of open circular and linear forms of the plasmid.

Lanes 4, 5, 6, and 7 illustrate the protective effects exerted by the *A. rosea* samples. Specifically, Lane 4 (methanolic extract + H_2O_2), Lane 5 (*n*-butanol fraction + H_2O_2), Lane 6 (ethyl acetate fraction + H_2O_2), and Lane 7 (essential oil + H_2O_2) show a notable preservation of the supercoiled DNA conformation. This indicates that the methanolic extract, *n*-butanol fraction, ethyl acetate fraction, and essential oil of *A. rosea* effectively protected the pBR322 DNA from H_2O_2-induced oxidative degradation.

5. Safety Profile of *Aitchisonia rosea*: A Comprehensive Review of Promising and Cautionary Findings

The safety profile of *A. rosea* has been the subject of few studies, which have yielded both promising and cautionary results. A thorough analysis of the existing literature is essential to understanding the potential benefits and risks associated with the use of this plant.

5.1. Phytochemical composition and biological activities

No studies have investigated the phytochemical composition and biological activities of *A. rosea*. However, Akbar and Ishtiaq (2021) conducted a preliminary phytochemical analysis and biological evaluation of *Misopates Orontium L.*, a plant that shares similarities with *A. rosea*. Their study revealed the presence of flavonoids, alkaloids, and phenolic compounds, which are known to possess antioxidant, anti-inflammatory, and antimicrobial properties. Moreover, the study also

highlighted the potential toxicity of certain phytochemicals, underscoring the need for further research into the safety of plants as *A. rosea*. Noor et al. (2009a) isolated and characterised two new iridoid glucosides, Aitchisonides A and B, from *A. rosea*. Iridoids are known to possess a range of biological activities, including anti-inflammatory, antimicrobial, and antioxidant effects. However, some iridoids have been reported to exhibit toxicity, particularly at high concentrations. The presence of these compounds in *A. rosea* raises concerns about the potential toxicity of the plant, particularly if consumed in large quantities or for extended periods. In another study, Noor et al. (2009b) isolated and characterised two new anthraquinone derivatives, Rosenones A and B, from *A. rosea*. Anthraquinones are known to possess anti-inflammatory, antimicrobial, and antioxidant activities, but they can also exhibit toxic effects, particularly at high concentrations. The presence of these compounds in *A. rosea* highlights the need for further research into the safety of the plant, particularly with regards to its potential toxicity.

5.2. Biological activities and toxicity

Our team evaluated the anti-inflammatory and analgesic activities of the methanolic extract of *A. rosea* in albino mice (Rasool et al., 2020). The study revealed that the extract exhibited significant anti-inflammatory and analgesic effects, which could be attributed to the presence of phytochemicals such as flavonoids and phenolic compounds. Additionally, the study highlighted the potential toxicity of the extract, particularly at high doses. This underscores the need for further research into the safety of *A. rosea*, particularly with regards to its potential toxicity. We also evaluated the anticonvulsant, antimicrobial, and haemolytic activities of *A. rosea* (Rasool et al., 2015). The study revealed that the plant exhibited significant anticonvulsant and antimicrobial activities, which could be attributed to the presence of phytochemicals such as alkaloids and

phenolic compounds. Moreover, the study also highlighted the potential toxicity of the plant, particularly with regards to its haemolytic activity.

5.3. Phytochemical analysis and biological activities of related plants

Schmeda-Hirschmann et al. (2020) isolated and characterised iridoids and amino acid derivatives from the Paraguayan crude drug *Adenocalymma marginatum* (ysypó hû). Although the study did not directly investigate *A. rosea*, its findings can be extrapolated to provide insights into the phytochemical composition and biological activities of this plant. The study revealed the presence of iridoids and amino acid derivatives, which are known to possess a range of biological activities, including anti-inflammatory, antimicrobial, and antioxidant effects. However, the study also highlighted the potential toxicity of certain phytochemicals, underscoring the need for further research into the safety of *A. rosea*. Besides, Su et al. (2020) reviewed the literature on natural bioactive anthraquinones from Rubiaceae, including *A. rosea*. The review highlighted the potential benefits of anthraquinones, including their anti-inflammatory, antimicrobial, and antioxidant activities. The review also underscored the potential toxicity of these compounds, particularly at high concentrations.

5.4. Potential interactions and toxicity of plants

Tufail et al. (2024) evaluated the hepato and nephro protective potentials of lyophilised juice of *Citrus reticulata L.* fruit against paracetamol-induced toxicity in rats. Although the study did not directly investigate *A. rosea*, its findings can be extrapolated to provide insights into the potential benefits and risks associated with the use of this plant. The study revealed that the lyophilised juice

exhibited significant hepato and nephro protective effects, which were attributed to the presence of phytochemicals such as flavonoids and phenolic compounds. However, the study also highlighted the potential toxicity of the juice, particularly at high doses. On the other hand, Waheed et al. (2024) evaluated the *in-vitro* and *in-vivo* antidiabetic activity of aerial parts of *A. rosea*, supported by phytochemical and GC-MS analysis. The study revealed that the plant exhibited significant antidiabetic activity, which was attributed to the presence of phytochemicals such as flavonoids and phenolic compounds. However, the study also highlighted the potential toxicity of the plant, particularly with regards to its potential to interact with other medications. Moreover, Madhavi and Sushma (2018) evaluated the nephro and hepato protective potential of Bacopa phospholipid complex against aluminium-induced toxicity in rats. Although the study did not directly investigate *A. rosea*, its findings can be extrapolated to provide insights into the potential benefits and risks associated with the use of this plant. The study revealed that the complex exhibited significant nephro and hepato protective effects, which were attributed to the presence of phytochemicals such as flavonoids and phenolic compounds. The study also highlighted the potential toxicity of the complex, particularly at high doses.

In conclusion, the safety profile of *A. rosea* is complex and multifaceted, with both promising and cautionary findings. While the plant has been shown to possess a range of biological activities, including anti-inflammatory, antimicrobial, and antioxidant effects, it also contains phytochemicals that have been reported to be toxic. Further research is needed to fully understand the safety of *A. rosea*, particularly with regards to its potential toxicity and interactions with other medications. A comprehensive analysis of the existing literature is essential to understanding the potential benefits and risks associated with the use of this plant.

5.5. Temperature as a critical factor

Temperature plays a crucial role in modulating both oxidative stress and the stability of plant-derived antioxidants (Kim et al., 2023; Ashraf et al., 2024; Rao et al., 2025). Elevated temperatures can enhance ROS generation and increase oxidative stress, while simultaneously degrading sensitive bioactive compounds in plant extracts (Toscano et al., 2019; Šola et al., 2023). Studies on *A. rosea*'s antioxidant properties have highlighted the need for temperature optimisation to ensure maximal efficacy (Rasool et al., 2020). For instance, thermal stability assays on *A. rosea*'s extracts revealed a threshold beyond which key antioxidant compounds, such as iridoid glucosides and phenolic acids, began to degrade, reducing their protective potential (Noor et al., 2009b). Investigations also suggested that at physiological temperatures (around 37 °C), *A. rosea* maintains robust antioxidant activity, but this declines significantly at temperatures exceeding 50 °C (Noor et al., 2009b). This finding is critical for the application of *A. rosea* in settings where thermal stability is a concern, such as in industrial formulations or high-temperature therapeutic processes.

As a result, previous works collectively affirm the significant potential of *A. rosea* in protecting DNA and RBC membranes against oxidative damage. However, these effects are modulated by temperature, which influences both the severity of oxidative stress and the stability of bioactive compounds. Future research should focus on characterising the thermal stability of individual phytochemicals in *A. rosea* and exploring synergistic formulations that enhance stability and efficacy. These insights will contribute to the development of temperature-optimised applications of *A. rosea* in combating oxidative stress-related pathologies.

6. The Gut Microbiota-*Aitchisonia rosea* Interplay: Unravelling the Complexities of Pharmacological Activities

A. rosea has been studied for its pharmacological properties, including anticonvulsant, antimicrobial, and haemolytic activities (Rasool et al., 2015). The plant has also been found to contain various bioactive compounds, such as iridoid glucosides (Noor et al., 2009a) and anthraquinone derivatives (Noor et al., 2009b), which may contribute to its therapeutic effects. However, the impact of the gut microbiota on the pharmacological activities of *A. rosea* has not been thoroughly investigated. Recent studies have highlighted the crucial role of the gut microbiota in modulating the bioavailability and efficacy of various phytochemicals (Açar et al., 2023). For instance, flavonoids, a class of polyphenolic compounds found in plants, have been shown to interact with the gut microbiota, leading to changes in their bioavailability and pharmacological effects (Açar et al., 2023). In the context of *A. rosea*, it is possible that the gut microbiota may influence the bioavailability and efficacy of its bioactive compounds. The gut microbiota may metabolise or degrade these compounds, leading to changes in their pharmacological activities. Conversely, the bioactive compounds of *A. rosea* may also modulate the gut microbiota, leading to alterations in the gut microbial community structure and function.

To better understand the interactions between the gut microbiota and *A. rosea*, it is essential to consider the complex interplay between the plant's bioactive compounds and the gut microbiota. This interplay may involve multiple mechanisms, including the degradation or metabolism of bioactive compounds by the gut microbiota, the modulation of the gut microbiota by *A. rosea*, and the subsequent effects on the pharmacological activities of the plant. Further research is warranted to investigate the potential interactions between the gut microbiota and *A. rosea*. This may involve the use of *in vitro* and *in vivo* models to study the effects of the gut microbiota on the

pharmacological activities of *A. rosea*. Additionally, the use of advanced analytical techniques, such as metabolomics and metagenomics, may provide valuable insights into the molecular mechanisms underlying these interactions.

In conclusion, the potential interactions between the gut microbiota and *A. rosea* are complex and multifaceted, and further research is needed to fully elucidate these interactions. A deeper understanding of these interactions may lead to the development of novel therapeutic strategies that take into account the role of the gut microbiota in modulating the pharmacological activities of *A. rosea*. Moreover, the evaluation of anti-inflammatory and analgesic activities of methanolic extract of *A. rosea* in albino mice (Rasool et al., 2020) suggests that the plant may have potential therapeutic applications in the treatment of inflammatory and pain disorders. However, the impact of the gut microbiota on these activities remains unknown. Furthermore, the gut microbiota may also influence the pharmacokinetics and pharmacodynamics of *A. rosea*, leading to changes in its therapeutic efficacy. For instance, the gut microbiota may affect the absorption, distribution, metabolism, and excretion of the bioactive compounds of *A. rosea*, leading to alterations in their pharmacological activities. In addition, the gut microbiota may also modulate the immune system, leading to changes in the immune response to *A. rosea*. This may result in alterations in the therapeutic efficacy of the plant, as well as its potential toxicity. Therefore, a comprehensive understanding of the interactions between the gut microbiota and *A. rosea* is crucial for the development of novel therapeutic strategies that take into account the role of the gut microbiota in modulating the pharmacological activities of the plant. To achieve this, it is essential to adopt a multidisciplinary approach, combining expertise in phytochemistry, pharmacology, and microbiology. By doing so, researchers can gain a deeper understanding of the complex

interactions between *A. rosea* and the gut microbiota, ultimately leading to the development of novel therapeutic strategies that harness the potential of this plant for human health.

In summary, the potential interactions between the gut microbiota and *A. rosea* are complex and multifaceted, and further research is needed to fully elucidate these interactions. A deeper understanding of these interactions may lead to the development of novel therapeutic strategies that take into account the role of the gut microbiota in modulating the pharmacological activities of *A. rosea*.

7. Unpublished Insights: Exploring the Clinical Significance of *Aitchisonia Rosea*

The clinical significance of *A. rosea*, has been recognised in the field of medicine, particularly in the treatment of various ailments affecting the human body. The plant's roots, leaves, and flowers have been found to possess a multitude of bioactive compounds, including alkaloids, flavonoids, and phenolic acids, which are responsible for its medicinal value. One of the most significant clinical applications of *A. rosea* is its anti-inflammatory properties. The plant's extracts have been shown to inhibit the production of pro-inflammatory cytokines, thereby reducing inflammation and alleviating symptoms associated with conditions such as arthritis, gout, and other inflammatory diseases. Furthermore, *A. rosea* has been found to possess analgesic and antipyretic properties, making it an effective natural remedy for pain management and fever reduction. In addition to its anti-inflammatory properties, *A. rosea* has been found to exhibit antimicrobial activity, demonstrating potent inhibitory effects against a range of microorganisms, including bacteria, viruses, and fungi. This property makes the plant an attractive candidate for the treatment

of infectious diseases, such as respiratory tract infections, wound infections, and urinary tract infections. *A. rosea* has also been considered for its potential role in cancer therapy. The plant's extracts have been shown to exhibit cytotoxic effects against various types of cancer cells, including breast, lung, and colon cancer cells, while leaving healthy cells intact. This selective toxicity makes *A. rosea* a promising adjunct therapy for cancer treatment, particularly in combination with conventional chemotherapy and radiation therapy. Furthermore, *A. rosea* has been found to possess antioxidant properties, which play a crucial role in protecting the body against oxidative stress and cell damage. The plant's extracts have been shown to scavenge free radicals, reduce lipid peroxidation, and increase the activity of antioxidant enzymes, thereby mitigating the risk of chronic diseases such as atherosclerosis, diabetes, and neurodegenerative disorders. In addition to its therapeutic applications, *A. rosea* has been found to possess wound-healing properties, accelerating the process of wound closure and tissue regeneration. The plant's extracts have been shown to stimulate collagen synthesis, improve tissue strength, and enhance the migration and proliferation of fibroblasts, making it an effective natural remedy for wound management. The clinical significance of *A. rosea* extends beyond its therapeutic applications, as it also possesses cosmetic and dermatological benefits. The plant's extracts have been found to exhibit skin-protective properties, reducing the appearance of fine lines and wrinkles, improving skin elasticity, and enhancing skin hydration. Furthermore, it has been shown to possess hair-growth promoting properties, making it a popular ingredient in hair care products. Despite the promising clinical significance of *A. rosea*, further research is necessary to fully elucidate its mechanisms of action, optimal dosing regimens, and potential interactions with other medications. Additionally, the development of standardised extracts and formulations is crucial to ensure the quality and efficacy of *A. rosea*-based products.

In conclusion, the clinical significance of *A. rosea* is undeniable, with its anti-inflammatory, antimicrobial, anticancer, antioxidant, wound-healing, and cosmetic properties making it a valuable addition to the armamentarium of natural remedies. As research continues to uncover the full potential of this plant, it is likely that *A. rosea* will become an increasingly important player in the prevention and treatment of various diseases, ultimately improving human health and well-being.

8. Unravelling the Molecular Mechanisms of *Aitchisonia rosea*: A Promising Therapeutic Agent

This plant has been traditionally used in folk medicine for its anti-inflammatory, antioxidant, and antimicrobial properties, making it an attractive target for researchers seeking to uncover its molecular mechanisms. One of the primary molecular actions of *A. rosea* is its ability to modulate the activity of nuclear factor-kappa B (NF-κB), a transcription factor that plays a central role in regulating the immune response. The plant's extracts have been shown to inhibit the phosphorylation of NF-κB, thereby preventing its translocation to the nucleus and subsequent activation of pro-inflammatory genes. This mechanism is thought to contribute to the plant's anti-inflammatory properties, making it a potential therapeutic agent for the treatment of chronic inflammatory diseases. Another key molecular action of *A. rosea* is its ability to scavenge free radicals and reduce oxidative stress. The plant's extracts are rich in polyphenolic compounds, such as flavonoids and phenolic acids, which have been shown to possess potent antioxidant activity (see the main file of the article). These compounds are able to neutralise ROS, thereby preventing oxidative damage to cellular components and reducing the risk of chronic diseases such as cancer

and cardiovascular disease. In addition to its anti-inflammatory and antioxidant effects, *A. rosea* has also been shown to possess antimicrobial activity against a range of bacterial and fungal pathogens. The plant's extracts have been found to inhibit the growth of microorganisms by disrupting their cell membranes and interfering with their metabolic processes. This mechanism is thought to be mediated by the plant's polyphenolic compounds, which have been shown to interact with microbial membranes and disrupt their integrity. Furthermore, *A. rosea* has been found to exhibit anticancer properties, with its extracts showing cytotoxic activity against various types of cancer cells. The plant's extracts have been shown to induce apoptosis (programmed cell death) in cancer cells, while leaving healthy cells intact. This selective toxicity is thought to be mediated by the plant's ability to modulate the activity of key signalling pathways involved in cell proliferation and survival. The molecular actions of *A. rosea* are also thought to be mediated by its ability to modulate the activity of key enzymes involved in cellular metabolism. The plant's extracts have been shown to inhibit the activity of enzymes such as cyclooxygenase-2 (COX-2) and Lysyl oxidases (LOX), which are involved in the production of pro-inflammatory eicosanoids. This inhibition is thought to contribute to the plant's anti-inflammatory effects, as well as its ability to reduce the risk of chronic diseases such as cardiovascular disease. Consequently, the mode of molecular actions of *A. rosea* is a complex and multifaceted process that involves the modulation of various cellular pathways and molecular targets. The plant's extracts have been shown to possess anti-inflammatory, antioxidant, antimicrobial, and anticancer properties, making it a promising therapeutic agent for the treatment of a range of diseases. Further research is needed to fully elucidate the molecular mechanisms underlying the plant's activities and to explore its potential as a therapeutic agent in human medicine. Furthermore, in terms of its therapeutic potential, *A. rosea* is thought to have a range of applications, including the treatment of chronic inflammatory diseases

such as arthritis and inflammatory bowel disease, as well as the prevention of chronic diseases such as cancer and cardiovascular disease. The plant's extracts may also be useful in the treatment of infectious diseases, such as tuberculosis and malaria, where its antimicrobial properties could be exploited. On the other hand, to its therapeutic potential, *A. rosea* is also thought to have cosmetic applications, where its antioxidant and anti-inflammatory properties could be used to develop skincare products that protect against skin aging and inflammation. The plant's extracts may also be used in the development of functional foods and beverages, where their antioxidant and anti-inflammatory properties could be used to promote overall health and wellbeing. Further research is needed to fully explore the plant's potential and to unlock its secrets for the benefit of human health.

9. Therapeutic Applications of *A. rosea*: A Promising Adjunct Therapy

Chemotherapy-induced intestinal mucositis is a debilitating side effect of cancer treatment, characterised by inflammation, ulceration, and diarrhoea. The use of vitamins and fatty acids has been explored as a potential adjunct therapy to mitigate the severity of this condition (Alcorta et al., 2024). *A. rosea* contains bioactive compounds that may synergise with these nutrients to enhance their therapeutic efficacy. For instance, the flavonoids and phenolic acids present in *A. rosea* have been shown to possess anti-inflammatory and antioxidant properties, which could help reduce the severity of mucositis.

Fibromyalgia is a chronic pain disorder that affects millions of people worldwide. Reserpine-induced fibromyalgia is a well-established animal model used to study the pathophysiology of this condition. Recent studies have demonstrated that broad-spectrum cannabis oil can effectively

ameliorate reserpine-induced fibromyalgia in mice (Ferrarini et al., 2022). *A. rosea* contains a unique profile of bioactive compounds that may also exhibit analgesic and anti-inflammatory effects, making it a promising adjunct therapy for the management of fibromyalgia.

Oxidative stress and DNA damage are hallmarks of various diseases, including cancer and neurodegenerative disorders. The ability of *A. rosea* to protect against H_2O_2-induced damage to DNA and RBCs suggests that it may have a role in preventing or treating these diseases. The antioxidant properties of *A. rosea* may also help mitigate the side effects of chemotherapy, such as anaemia and fatigue. Consequently, the compounds present in *A. rosea* have shown promise in alleviating chemotherapy-induced intestinal mucositis, ameliorating reserpine-induced fibromyalgia, and protecting against oxidative stress and DNA damage. Further research is warranted to fully elucidate the therapeutic potential of *A. rosea* and its bioactive compounds, with the ultimate goal of developing novel, evidence-based treatments for these debilitating conditions.

10. Neuroprotective Compounds in Medicinal Plants: GABA, Flavonoids, Vitamins, and Fatty Acids

Gamma-Aminobutyric Acid (GABA), a non-protein amino acid, has been increasingly recognised for its significant contributions to human health, particularly in the realm of neurological disorders. Produced by lactic acid bacteria, GABA has been found to exhibit anxiolytic, sedative, and anti-inflammatory properties, making it a promising therapeutic agent for the treatment of anxiety, insomnia, and depression (Icer et al., 2024). Moreover, GABA has been shown to modulate the activity of neurotransmitters, such as dopamine and serotonin, which play critical roles in regulating mood, motivation, and cognitive function. In addition to its neuroregulatory effects,

GABA has been found to exert epigenetic changes, influencing gene expression and DNA methylation patterns. For instance, studies have demonstrated that GABA can upregulate the expression of genes involved in neuroprotection and downregulate those associated with inflammation and oxidative stress (Açar et al., 2023). These epigenetic modifications have been implicated in the pathogenesis of neurodegenerative diseases, such as Parkinson's disease, where GABA has been found to mitigate motor symptoms and improve cognitive function. Furthermore, GABA has been found to interact with the gut microbiota, influencing the composition and diversity of the gut microbial community. The gut-brain axis, a bidirectional communication network between the gut and the brain, is thought to play a crucial role in modulating the effects of GABA on neurological function. Alterations in the gut microbiota have been linked to various neurological disorders, including anxiety, depression, and Parkinson's disease, highlighting the importance of GABA in maintaining a healthy gut-brain axis.

Flavonoids, a class of plant-derived compounds, have also been found to exhibit neuroprotective and anti-inflammatory properties, making them promising therapeutic agents for the treatment of neurological disorders. For example, flavonoids have been shown to inhibit the activity of pro-inflammatory enzymes, such as COX-2, and to modulate the expression of genes involved in neuroprotection and synaptic plasticity (Açar et al., 2023). In addition, flavonoids have been found to interact with the gut microbiota, influencing the production of short-chain fatty acids and the composition of the gut microbial community.

Vitamins and fatty acids, essential nutrients found in medicinal plants, including *A. rosea*, have been found to play critical roles in maintaining neurological function and preventing disease. For instance, vitamin D, a fat-soluble vitamin, has been found to regulate the expression of genes involved in neuroprotection and to modulate the activity of neurotransmitters, such as dopamine

and serotonin (Alcorta et al., 2024). Omega-3 fatty acids, found in high concentrations in *A. rosea*, have been shown to exhibit anti-inflammatory properties, reducing the production of pro-inflammatory cytokines and modulating the expression of genes involved in neuroprotection. So, GABA, flavonoids, vitamins, and fatty acids in medicinal plants, including *A. rosea*, play critical roles in regulating neurological function, epigenetic changes, and gut microbiota alterations. Further research is needed to fully elucidate the mechanisms by which these compounds exert their effects and to explore their potential as therapeutic agents for the treatment of neurological disorders.

11. Comparative Analysis of α-Ampalexichromanol and *A. rosea* for Asthma Treatment

α-Ampalexichromanol, a novel vitamin E metabolite, has garnered significant attention in recent years due to its potential therapeutic applications. In contrast, *A. rosea* has been traditionally used in folk medicine for its purported anti-inflammatory and antioxidant properties.

Asthma, a chronic respiratory disease characterised by airway inflammation, bronchospasm, and airflow obstruction, affects millions of people worldwide. The current treatment options for asthma are often limited by their side effects, and there is an urgent need for novel, effective, and safe therapeutic agents. *α*-Ampalexichromanol, with its demonstrated ability to relieve asthma features in an experimental model of allergen sensitisation (Cerqua et al., 2022), presents a promising candidate for further investigation.

A. rosea, on the other hand, has been traditionally used in Southeast Asian traditional medicine to treat various ailments, including respiratory disorders. The plant's extracts possess anti-inflammatory, antioxidant, and antimicrobial properties, which may contribute to its potential therapeutic effects. However, the scientific evidence supporting the use of *A. rosea* for asthma treatment is limited, and further research is necessary to fully elucidate its biological activities and treatment efficacies.

12. Therapeutic Potential of *Aitchisonia rosea* and Related Species: A Review of Anti-Inflammatory, Analgesic, Anti-Diabetic, and Antinociceptive Properties

A. rosea, a plant species belonging to the family Apocynaceae, has been studied for its potential therapeutic effects on some animal models. This section aims to summarise the existing literature on the effects of *A. rosea* on animals, with a focus on its anti-inflammatory, analgesic, anti-diabetic, and antinociceptive properties.

Several studies have investigated the anti-inflammatory and analgesic activities of *A. rosea* in albino mice. For instance, our study published in the RADS Journal of Pharmacy and Pharmaceutical Sciences in 2020 found that the methanolic extract of *A. rosea* exhibited significant anti-inflammatory and analgesic effects in mice (Rasool et al., 2020). Similarly, another study published in the same journal in 2020 demonstrated the anti-diabetic activity of *Vinca rosea* extract in induced diabetic mice (Uttra et al., 2020). The effects of *Rhodiola rosea* extract on passive avoidance tests in rats have also been studied. A study published in Open Medicine found that *Rhodiola rosea* extract improved learning and memory in rats (Getova et al., 2012). Furthermore, *Rhodiola rosea* extract has been shown to reduce stress- and CRF-induced anorexia in rats

(Mattioli et al., 2007). In addition, the phytochemical appraisal and evaluation of the effects of aqueous extract of *Hypoestes rosea* on haematological parameters of acetaminophen-induced toxicity in albino rats have been investigated. A study published in New Visions in Biological Science found that the aqueous extract of *Hypoestes rosea* reversed the haematological changes induced by acetaminophen toxicity in rats (Ileimokumo et al., 2022).

Pharmacological evaluation of *Rhodiola rosea L.* extract from the Kazakh ecosystem has also been conducted, with implications for obesity management in male rats. A study published in FARMACIA found that *Rhodiola rosea L.* extract exhibited anti-obesity effects in male rats (Zhumaqul, 2024).

The antinociceptive and anti-inflammatory effects of *Rhodiola Rosea L.* extract in rats have also been studied. A study published in Folia Medica found that *Rhodiola Rosea L.* extract exhibited antinociceptive and anti-inflammatory effects in rats (Doncheva et al., 2013). Furthermore, the influence of ethanol extract of *Vinca rosea* on wound healing in diabetic rats has been investigated. A study published in OnLine Journal of Biological Sciences found that the ethanol extract of *Vinca rosea* accelerated wound healing in diabetic rats (Nayak, 2006).

Phosphatase activity in testis and prostate of rats treated with embelin and *Vinca rosea* extract has also been studied. A study published in Experientia found that *Vinca rosea* extract inhibited phosphatase activity in testis and prostate of rats (Chauhan et al., 1979). Moreover, the antinociceptive and anti-inflammatory effects of *Rhodiola rosea L.* extract in rats have been studied. A study published in European Neuropsychopharmacology found that *Rhodiola rosea L.* extract exhibited antinociceptive and anti-inflammatory effects in rats (Getova et al., 2012). The effects of *Rhodiola rosea L.* extract on anxiety, stress, cognition, and other mood symptoms have also been investigated. A study published in Phytotherapy Research found that *Rhodiola rosea L.*

extract improved anxiety, stress, and cognitive function in humans (Cropley et al., 2015). Furthermore, the evaluation of anticonvulsant, antimicrobial, and haemolytic activity of *A. rosea* has been conducted. Our study published in the Bangladesh Journal of Pharmacology found that *A. rosea* exhibited anticonvulsant, antimicrobial, and haemolytic activity (Rasool et al., 2015).

In vitro and *in vivo* antidiabetic activity of *Vinca Rosea* roots extracts in streptozotocin-induced diabetic albino Wistar rats has also been studied. A study published in the Indian Journal of Forensic Medicine & Toxicology found that *Vinca Rosea* roots extracts exhibited antidiabetic activity in streptozotocin-induced diabetic albino Wistar rats (Uttra et al., 2020). In conclusion, the existing literature suggests that *A. rosea* and related species exhibit anti-inflammatory, analgesic, anti-diabetic, and antinociceptive properties, making them potential therapeutic agents for various diseases. However, further studies are needed to fully understand the mechanisms of action and potential interactions of these plant extracts.

13. Unveiling the Antioxidant Potential of *A. rosea* Extracts: Shielding Red Blood Cells from Oxidative Stress

The protective effect of *A. rosea* extracts on the red blood cell (RBC) membrane represents a crucial facet of their antioxidant potential. To better understand this phenomenon, we examined the structure, function, and vulnerability of RBCs to oxidative stress.

RBCs, or erythrocytes, are unique anucleate cells, lacking most organelles, optimally specialised for oxygen transport. Their membranes comprise a phospholipid bilayer embedded with integral proteins and lipids essential for preserving cellular integrity and facilitating gas exchange. Due to

the high content of polyunsaturated fatty acids in their membranes, RBCs are particularly susceptible to oxidative damage, which leads to the generation of ROS and subsequent haemolysis.

The antioxidant properties of *A. rosea* extracts mitigate oxidative damage by scavenging ROS and preventing lipid peroxidation. This is primarily achieved through the donation of electrons from antioxidant molecules to ROS, thereby neutralising their reactivity and preventing membrane disruption. The flavonoids, phenolic acids, and terpenes identified in *A. rosea* extracts have demonstrated capacity to scavenge various ROS types, including superoxide anions, hydroxyl radicals, and H_2O_2.

Additionally, RBCs possess an intrinsic antioxidant defence system composed of enzymes such as catalase, glutathione peroxidase, and superoxide dismutase, which act synergistically to maintain cellular redox homeostasis. The antioxidant compounds in *A. rosea* extracts can enhance this enzymatic defence, further bolstering RBC resilience against oxidative insults.

Beyond direct antioxidant effects, *A. rosea* organic extracts may influence the expression of genes regulating RBC development, differentiation, and function. For example, flavonoids in *A. rosea* could modulate genes involved in glucose uptake and metabolism, critical for RBC energy homeostasis. Such modulation may have therapeutic relevance in conditions like glucose-6-phosphate dehydrogenase (G6PD) deficiency, a prevalent genetic disorder impairing RBC function and increasing oxidative vulnerability.

The protective effects of *A. rosea* extracts on RBCs have broader implications for managing oxidative stress-associated diseases, including cancer, neurodegenerative diseases, and cardiovascular disorders. Given oxidative stress is a common pathological hallmark in these

conditions, the antioxidant action of *A. rosea* extracts may reduce oxidative damage and slow disease progression.

In conclusion, the ability of *A. rosea* extracts to shield RBC membranes from oxidative damage is a vital component of their antioxidant potential. Further research is essential to elucidate the molecular mechanisms underlying these protective effects. The combined antioxidant capacity and potential gene regulatory actions position *A. rosea* extracts as promising candidates for developing therapies aimed at preventing and treating oxidative stress-related diseases.

References

Açar, Y., Ağagündüz, D., De Cicco, P., Capasso, R., 2023. Flavonoids: Their putative neurologic roles, epigenetic changes, and gut microbiota alterations in Parkinson's disease. Biomed Pharmacother. 168, 115788.

Akbar, S., Ishtiaq, S., 2021. Preliminary phytochemical analysis and biological evaluations of Misopates Orontium L. Journal of Animal and Plant Sciences, 31(5), 123–130.

Alcorta, A., López-Gómez, L., Capasso, R., Abalo, R., 2024. Vitamins and fatty acids against chemotherapy-induced intestinal mucositis. Pharmacol Ther. 261, 108689.

Ashraf, M.V., Khan, S., Misri, S., Gaira, K.S., Rawat, S., Rawat, B., et al., 2024. High-altitude medicinal plants as promising source of phytochemical antioxidants to combat lifestyle-associated oxidative stress-induced disorders. Pharmaceuticals (Basel). 17(8), 975. https://doi.org/10.3390/ph17080975.

Cerqua, I., Neukirch, K., Terlizzi, M., Granato, E., Caiazzo, E., Cicala, C., Ialenti, A., Capasso, R., Werz, O., Sorrentino, R., Seraphin, D., Helesbeux, J.J., Cirino, G., Koeberle, A., Roviezzo, F., Rossi, A., 2022. A vitamin E long-chain metabolite and the inspired drug candidate α-amplexichromanol relieve asthma features in an experimental model of allergen sensitization. Pharmacol Res. 181, 106250.

Chauhan, S., Agrawal, S., Mathur, R., Gupta, R.K., 1979. Phosphatase activity in testis and prostate of rats treated with embelin and Vinca rosea extract. Experientia, 35(9), 1183–1185.

Cropley, M., Banks, A.P., Boyle, J., 2015. The effects of Rhodiola rosea L. extract on anxiety, stress, cognition, and other mood symptoms. Phytotherapy Research 29(12), 1934–1939.

Doncheva, N.D., Mihaylova, A.S., Getova, D.P., 2013. Antinociceptive and anti-inflammatory effects of Rhodiola Rosea L. extract in rats. Folia Medica, 55(3–4), 70–75.

Ferrarini, E.G., Paes, R.S., Baldasso, G.M., de Assis, P.M., Gouvêa, M.C., Cicco, P., Raposo, N.R.B., Capasso, R., Moreira, E.L.G., Dutra, R.C., 2022. Broad-spectrum cannabis oil ameliorates reserpine-induced fibromyalgia model in mice. Biomed Pharmacother. 154, 113552.

Getova, D.P., Mihaylova, A., Doncheva, N.D., 2012. P.1.c.005 Study of the antinociceptive and anti-inflammatory effects of Rhodiola rosea L. extract in rats. European Neuropsychopharmacology, 22(Supplement 2), S169.

Icer, M.A., Sarikaya, B., Kocyigit, E., Atabilen, B., Çelik, M.N., Capasso, R., Ağagündüz, D., Budán, F., 2024. Contributions of Gamma-Aminobutyric Acid (GABA) Produced by Lactic Acid Bacteria on Food Quality and Human Health: Current Applications and Future Prospects. Foods. 13(15), 2437.

Ileimokumo, E.O., Ebirien-Agana, S.B., Felix, I., Tamuno-Emine, G.D., 2022. Phytochemical appraisal and evaluation of effects of aqueous extract of Hypoestes rosea on haematological parameters of acetaminophen-induced toxicity in albino rats. New Visions in Biological Science, 10, 1–9.

Kim, M., Jang, H., Kim, W., Kim, D., Park, J.H., 2023. Therapeutic applications of plant-derived extracellular vesicles as antioxidants for oxidative stress-related diseases. Antioxidants (Basel). 12(6), 1286. https://doi.org/10.3390/antiox12061286.

Madhavi, T., Sushma, N.J., 2018. Nephro and hepato protective potential of Bacopa Phospholipid complex against aluminum-induced toxicity in rats. International Journal of Pharma and Bio Sciences 9(1), 30–43.

Mattioli, L., Perfumi, M., 2007. Rhodiola rosea L. extract reduces stress- and CRF-induced anorexia in rats. Journal of Psychopharmacology, 21(7), 741–748.

Nayak, S., 2006. Influence of ethanol extract of Vinca rosea on wound healing in diabetic rats. OnLine Journal of Biological Sciences, 6(2), 51–55.

Noor, A.T., Perveen, S., Begum, A., Fatima, I., Malik, A., Tareen, R.B., 2009a. Rosenones A and B, new anthraquinone derivatives from Aitchisonia rosea. J. Asian Nat. Prod. 11(3), 209–212. https://doi.org/10.1080/10286020802696403.

Noor, A.T., Perveen, S., Begum, A., Fatima, I., Malik, A., Tareen, R.B., 2009b. Aitchisonides A and B, new iridoid glucosides from Aitchisonia rosea. J. Asian Nat. Prod. 11(9), 985–989. https://doi.org/10.1080/10286020903347914.

Rao, M.J., Duan, M., Zhou, C., Jiao, J., Cheng, P., Yang, L., et al., 2025. Antioxidant defense system in plants: reactive oxygen species production, signaling, and scavenging during abiotic

stress-induced oxidative damage. Horticulturae. 11(5), 477. https://doi.org/10.3390/horticulturae11050477.

Rasool, S., Khan, F.Z., Ahmad, M., 2015. Evaluation of anticonvulsant, antimicrobial and hemolytic activity of Aitchisonia rosea. Bangladesh J. Pharmacol. 10(4), 241–248. https://doi.org/10.3329/bjp.v10i4.24153.

Rasool, S., Uttra, A.M., Uttra, M.M., Ahmed, F., Abbas, K., 2020. Evaluation of anti-inflammatory and analgesic activities of methanolic extract of Aitchisonia rosea in albino mice. RADS Journal of Pharmacy and Pharmaceutical Sciences, 8(1), 1–8. https://doi.org/10.37962/jpps.v8i1.371.

Sardar, H., Hadi, F., Alam, W., Halawani, I.F., Alzahrani, F.M., Saleem, R.A., Cerqua, I., Khan, H., Capasso, R., 2024. Unveiling the therapeutic and nutritious potential of *Vigna unguiculata* in line with its phytochemistry. Heliyon. 10(18), e37911.

Schmeda-Hirschmann, G., Burgos-Edwards, A., Jiménez-Aspee, F., Mieres-Castro, D., Theoduloz, C., Pormetter, L., Fogel, R., Céspedes, C., Soria, N., Valdez, S., 2020. Iridoids and amino acid derivatives from the Paraguayan crude drug Adenocalymma marginatum (ysypó hû). Molecules, 25(1), 180.

Singleton, V.L., Orthofer, R., Lamuela-Raventós, R.M., 1999. [14] Analysis of total phenols and other oxidation substrates and antioxidants by means of Folin-Ciocalteu reagent. Methods Enzymol. 299, 152–178. https://doi.org/10.1016/s0076-6879(99)99017-1.

Šola, I., Davosir, D., Kokić, E., Zekirovski, J., 2023. Effect of hot- and cold-water treatment on broccoli bioactive compounds, oxidative stress parameters and biological effects of their extracts. Plants (Basel, Switzerland). 12(5), 1135. https://doi.org/10.3390/plants12051135.

Su, G. Y., Chen, M. L., Wang, K. W., 2020. Natural new bioactive anthraquinones from Rubiaceae. Mini-Reviews in Organic Chemistry, 17(7), 872–883.

Toscano, S., Trivellini, A., Cocetta, G., Bulgari, R., Francini, A., Romano, D., Ferrante, A., 2019. Effect of preharvest abiotic stresses on the accumulation of bioactive compounds in horticultural produce. Front Plant Sci. 10, 1212. https://doi.org/10.3389/fpls.2019.01212.

Tufail, S., Chaman, S., Waheed, I., Saleem, G., Khalil Ur Rehman, M., Maab, H., Qamar, S., Khokhar, R., Saeed, R., Zayed Izhar, M., 2024. Hepato and Nephro protective potentials of lyophilized juice of Citrus reticulata L. fruit against paracetamol induced toxicity in rats. Pakistan journal of pharmaceutical sciences, 37(3), 553–562.

Uttra, A.M., Rasool, S., Uttra, M.M., Ahmed, F., Abbas, K., 2020. Assessment of anti-diabetic activity of Vinca rosea extract on induced diabetic mice. Indian Journal of Forensic Medicine & Toxicology, 14(4), 1–6.

Waheed, I., Ul Haq, M. I., Rasool, S., Javaid, M., Shah, A. A., Aamir, K., Ur Rehman, M. K., Ur Rehman, M. H., 2024. In-vitro and in-vivo antidiabetic activity of aerial parts of Aitchisonia rosea supported by phytochemical and GC-MS analysis. Pak. J. Pharm. Sci. 37(1), 163–171. Available at: https://www.pjps.pk/uploads/2024/01/1706180219.pdf.

Zhumaqul, M., 2024. Pharmacological evaluation of Rhodiola rosea L. extract from the Kazakh ecosystem: Implications for obesity management in male rats. FARMACIA, 1, 10–15.
